# Management of traumatic dental injuries: a survey of paediatric emergency department health professionals

**DOI:** 10.1136/bmjpo-2022-001740

**Published:** 2023-03-22

**Authors:** Nathalie Gallichan, Sondos Albadri, Francine Watkins, Fadi Jarad, Shrouk Messahel, Stuart Hartshorn, Laura Gartshore

**Affiliations:** 1 Paediatric Dentistry Department, School of Dentistry, University of Liverpool, Liverpool, UK; 2 Paediatric Dentistry Department, Alder Hey Children's NHS Foundation Trust, Liverpool, UK; 3 University of Liverpool Faculty of Health and Life Sciences, Liverpool, UK; 4 Restorative Department, School of Dentistry, University of Liverpool, Liverpool, UK; 5 Paediatric Emergency Department, Alder Hey Children's NHS Foundation Trust, Liverpool, UK; 6 Emergency Department, Birmingham Children's Hospital, Birmingham, UK; 7 Birmingham Clinical Trials Unit, University of Birmingham, Birmingham, UK

**Keywords:** Dentistry, Data Collection

## Abstract

**Objective:**

To assess paediatric emergency department (PED) health professionals’ confidence, experience and awareness in managing traumatic dental injuries (TDIs).

**Design:**

A cross-sectional online survey.

**Setting:**

PED at Alder Hey Children’s Hospital and Birmingham Children’s Hospital.

**Results:**

94 ED health professionals responded. One-third of responders (n=26) encounter children with dental trauma daily or weekly. TDI teaching during undergraduate training was received by 13% (n=12) of responders, and 32% (n=30) had never received training. Responders thought they would benefit from online resources and regular teaching on paediatric TDIs, in addition to an easy-to-use decision-making tool to signpost families.

ED health professionals’ confidence in giving advice to families following a TDI, and in recognising types of TDIs, was notably low; −79 and −76 Net Promotor Score, respectively.

Responders’ awareness of how to recognise and manage TDIs was varied. Majority were aware of the need to attempt to reimplant an avulsed permanent tooth, and the need to refer a child presenting with a complex permanent tooth injury to the oncall dentist. However, very few responders commented on the importance of follow-up. Responders also raised concerns about the lack of dental services to treat TDIs in children.

**Conclusions:**

There is a need to enhance dental trauma teaching for all ED health professionals who encounter TDIs to increase their confidence and enable them to triage and advise patients appropriately. Additionally, increased signposting for families to the appropriate service could in turn improve outcomes and experience for children who experience a TDI.

**WHAT IS ALREADY KNOWN ON THIS TOPIC**

- There is a lack of dental teaching within undergraduate medical programmes, and often no teaching on dental trauma despite how commonly presents to medical health professionals.
- Timely acute management of dental trauma has a direct impact on the prognosis of an injured tooth.

**WHAT THIS STUDY ADDS**

- There is a demand to develop an easy-to-use decision-making tool for health professionals working in a paediatric emergency department.
- There is a need to signpost families towards the most efficient and effective place to help manage dental trauma.
- Health professionals working in paediatric emergency departments would benefit from online resources and regular teaching on paediatric dental trauma.

**HOW THIS STUDY MIGHT AFFECT RESEARCH, PRACTICE OR POLICY**

- Initiate discussions within regional Managed Clinical Networks regarding paediatric acute trauma pathways.
- Creation of a formal teaching element and resources on paediatric dental trauma for health professionals working in paediatric emergency departments.
- Encourage further research across the UK into paediatric acute dental trauma pathways.

## Introduction

Paediatric emergency departments (PEDs) are increasingly being used as providers of dental care within the UK.^1 2^ Dental presentations predominantly include those attending with dental caries or traumatic dental injuries (TDIs). It is estimated that 40% of children experience a TDI to their primary teeth and up to one-third of children sustain trauma to their permanent teeth.^3–6^ If TDIs were included in the world’s most frequent diseases, it could rank fifth.^7^

TDIs can usually be acutely managed by a general dental practitioner (GDP), however, families may choose to attend emergency departments (EDs) where they are triaged and assessed by ED health professionals. The optimal management of TDIs in the ED relies on a child being appropriately triaged, seeking advice from the oncall dentist if required, and appropriate advice being given. Some ED units may not have an oncall dentist. Inappropriate management and advice may lead to undesirable consequences including damage to developing permanent tooth, pulpal necrosis leading to pain and dental abscess, or resorption and ultimate loss of a permanent tooth.^8^

The provision of oral health education in undergraduate medical programmes is reported to be minimal, and TDI management is often non-existant.^9 10^ However, ED doctors are required to have knowledge of dental trauma as part of their specialty training.^11^ Studies have reported that medical health professionals’ knowledge of TDI management is lacking. Most studies focused on avulsion management and many are not generalisable to the UK national health service’s provision of emergency care.^12–19^ The study was designed to assess PED clinician experience, confidence and awareness of TDIs. Primary objectives were to determine clinician confidence in assessing and managing TDIs in children. Secondary objectives were to identify the training and education clinicians have received, to identify PED clinician awareness of resources which aid TDI management, and investigate how they manage TDIs.

## Method

### Study design, sample and distribution

The study was undertaken as a cross-sectional survey, distributed in November 2020 using convenience sampling, inviting all ED health professionals working in two of the largest PEDs to participate: Alder Hey Children’s Hospital (AHH) and Birmingham Children’s Hospital (BCH). AHH cares for 330 000 children across the North West of England and North Wales each year. BCH cares for 270 600 children across the West Midlands and mid Wales each year.

The target sample size was total number of health professionals who worked across the two PEDs (n=150). All members of staff who may triage, provide advice or manage children with a TDI were included and invited to participate, including ED doctors, nurses and nurse practitioners. There were no exclusions.

Participation was voluntary and opt-in. A prenotification email was sent to health professionals 1 week in advance to brief the target population of the study, sent via a PED Consultant identified at each unit. A reminder email was sent 2 weeks later. Four weeks after the initial email, the survey closed.

### Survey design and content

A self-administered online questionnaire was designed to gather quantitative and qualitative data, a copy can be found in the appendices. The anonymous questionnaire was constructed using Microsoft Forms and informed by literature review. All responses were confidential, untracked and IP addresses were not stored. The survey has been pretested for content and face validity using face-to-face feedback with a paediatric dentist clinicians and researchers and subsequently piloted among a convenience sample of clinicians who have experience working in ED to further inform development. Written and verbal feedback was encouraged and time to complete the questionnaire was recorded.

Responder demographics and experience in dental trauma management was collected and analysed using descriptive statistics. A total of 21 questions were asked including open and closed questions, Likert scales and theoretical clinical vignettes which were designed to assess TDI awareness, responders’ confidence and pragmatic decision making. Analysis was undertaken using Microsoft Forms and Microsoft Excel (2019). Confidence was assessed using a Likert scale and presented using a Net Promoter Score (NPS), which provides a number from −100 to 100 calculated by subtracting the percentage of detractors from the percentage of promoters, and excludes the passive responses. Qualitative answers were analysed and grouped into recurring answers and also into themes. All questions were compulsory in order to complete and submit the survey, therefore there was no missing data. The CROSS checklist was used when writing the report.^20^

## Results

### ED health professionals’ roles and experience

Of 94 responders, the majority were doctors (n=49), and 45 responders were nursing staff (table 1). 64.9% identified themselves as working in the North West and 35.1% from the West Midlands, respectively. Experience of working in PED was relatively evenly split; <1 year (n=23), 1–5 years (n=23), 5–10 years (n=21) and >10 years (n=27). Majority of responders encountered children with dental trauma a few-times-a-month (n=51), 23 responders encounter TDIs weekly, 3 responders encounter TDIs in children daily and 17 responders encounter TDIs a few times a year. There were no missing data, all questions were required to complete the questionnaire. Overall response rate was 63%.

**Table 1:** Clinician role within the PED

Grand total	94
Nurse	23
Consultant	21
Trainee doctor/clinical fellow (middle grade/registrar)	12
Trainee doctor/clinical fellow (junior level- core trainee/SHOr equivalent/GP trainee)	12
Advanced nurse/clinical practitioner (ANP)	11
Emergency nurse practitioner	7
Specialty doctor/associate specialist	4
Physician associate	2
Healthcare assistant	1
ANP trainee	1

GP, General Practitioner; PED, Paediatric Emergency Department; SHO, Senior House Officer.

### Teaching and education received on dental trauma

**Figure 1:**
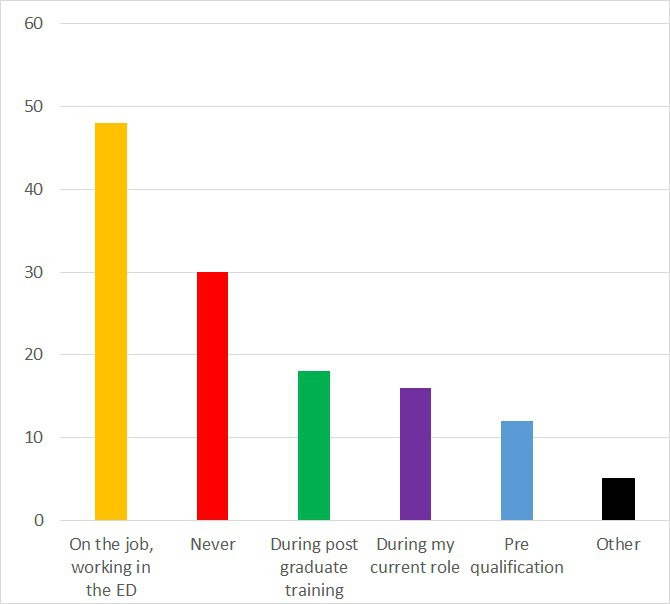
Teaching received on dental trauma. ED, emergency department.

**Figure 2:**
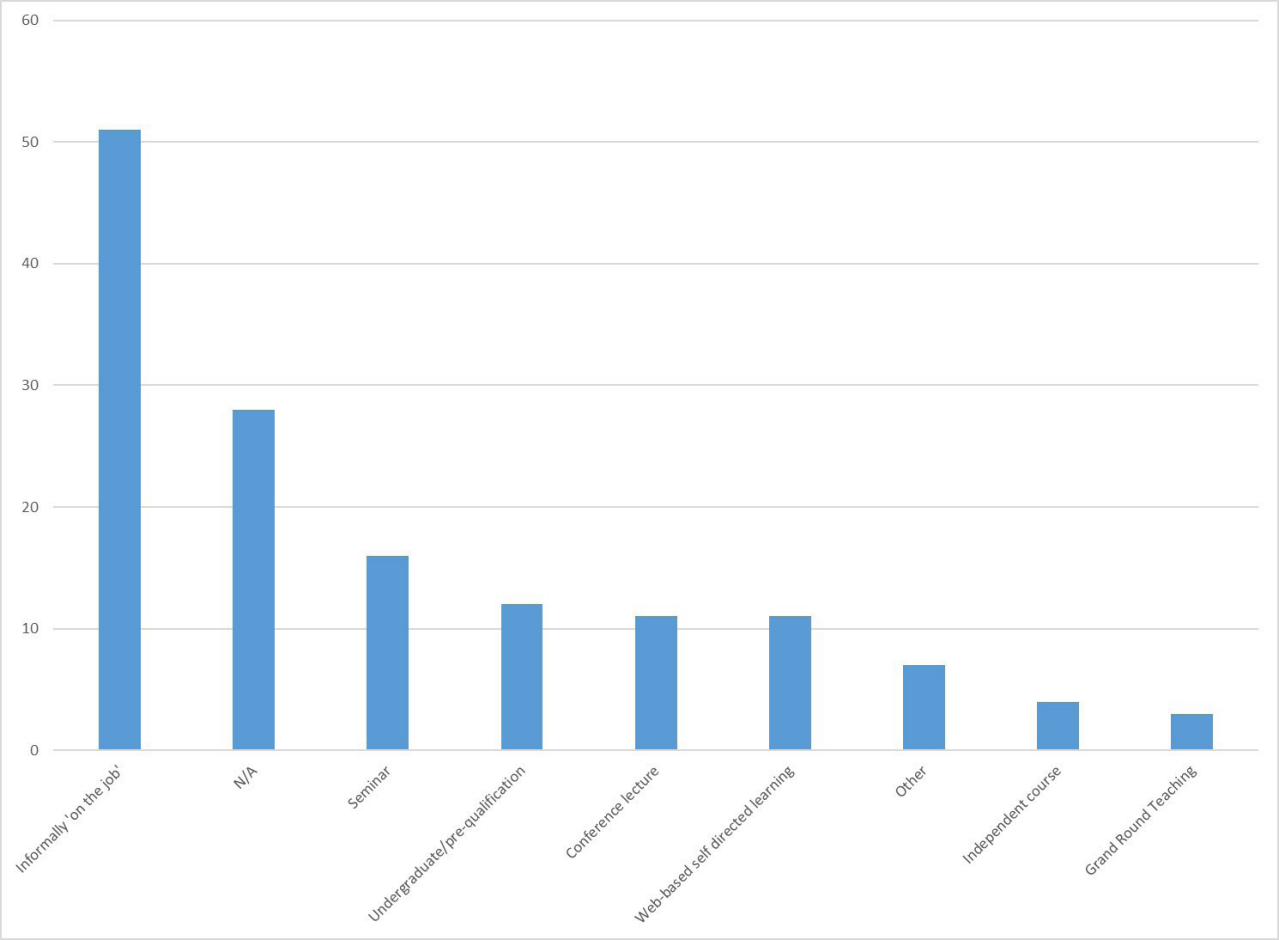
Format in which dental trauma teaching has been received. N/A, not applicable.

One-third of responders, (n=30) never received teaching about TDIs. Eighteen received teaching during postgraduate training, and twelve during undergraduate training (figure 1). The majority, (n=48), reported training ‘on the job, while working in the PED’. Figure 2 demonstrates the format of the teaching received.

The majority of responders, (n=63) reported they never used a TDI decision-making pathway in their department; 19 responders sometimes used one, and 12 responders regularly used one. Those who never used a TDI decision-making pathway had lower confidence scores than those who did. One-third of responders used resources including the dental trauma guide website,^21^ ‘internet’, teaching notes or an ED handbook to aid TDI recognition.

### Confidence

**Figure 3:**
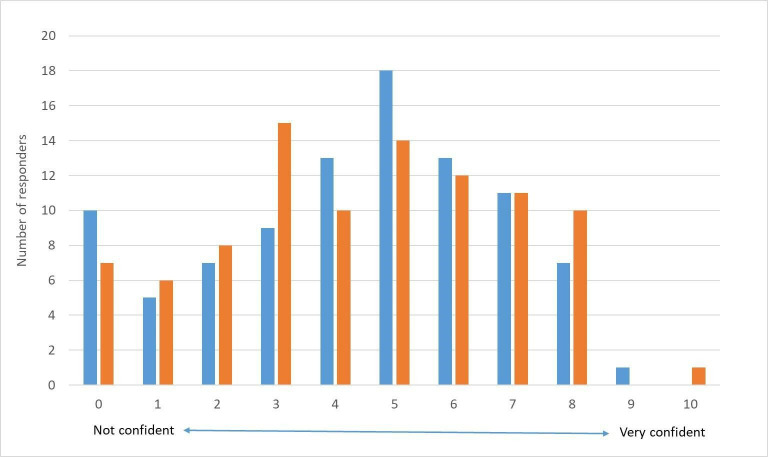
Responders' confidence in management of dental trauma in children. Blue=confidence in giving advice to children/parents following a dental trauma. Orange=confidence in recognising different types of trauma.

When providing advice to families following a TDI, and in recognising types of TDIs, responder’s confidence was notably low; −79 and −76 NPS, respectively. A total of 77%–80% of responders stated their confidence was less than 6/10 (figure 3). When subanalysed, consultants had the most confidence, followed by ANPs, with lowest confidence among physician associates. Trainee doctors and middle grades had notably lower confidence compared with ED consultants.

### Awareness of dental trauma and vignette responses

**Figure 4:**
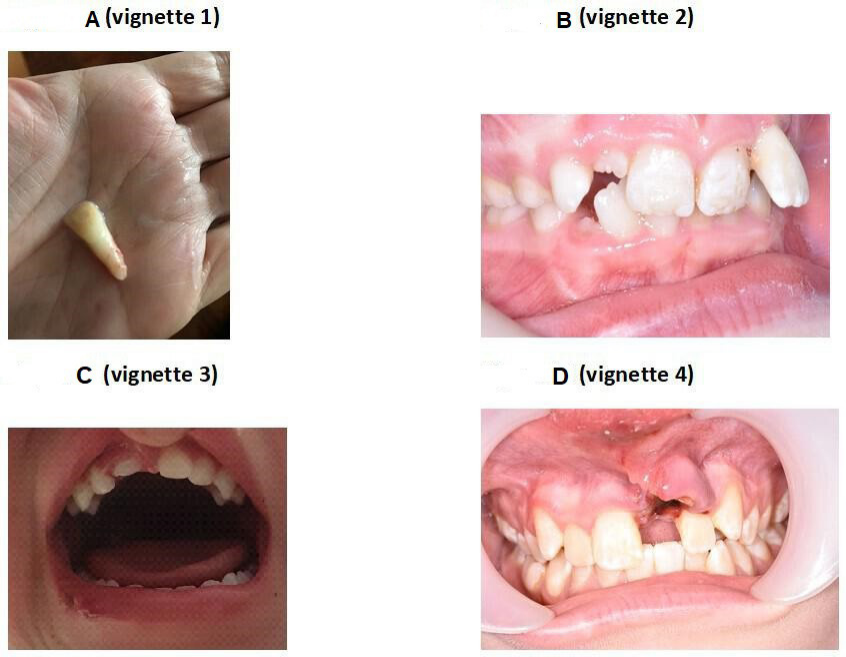
Photos used in each vignette.

Thematic analysis of the responders comments to the vignettes (V1–4), including figure 4A–D, led to the identification of the following themes:

- Oral maxillofacial surgery (OMFS) referral and utilisation for TDIs.
- Mixed awareness diagnosing and management of TDIs.
- Lack of confidence in managing TDIs.
- Confusion over recognising primary or permanent teeth.
- Limited follow-up advise provided.

When confronted with a complex intrusion and fracture injury in a permanent tooth (V4), 72 responders would refer to OMFS, compared with 22 who would refer for a primary tooth luxation injury (V3). V3 also elicited some responders (n=7) to refer to plastics or ENT.

Overall, 85 responders (90.4%) would advise to reimplant the avulsed tooth (V1) or place the tooth in an appropriate medium. In V1 and V2, 10 responders (10.6%) mentioned splinting the injured tooth, in V4, no responders mentioned splinting. Responders highlighted a lack of confidence in reimplanting a tooth, commenting *‘…Would not feel confident in placing tooth in situ also not fully sure if it is appropriate.’* Two responders would not provide any management for V1 except reassurance.

There was awareness from some responders that V3 was an intruded primary tooth (n=12) and some also displayed awareness that it ‘*may have impacted or damaged the permanent tooth’*. V2 and V4 were more complex TDIs, with less responders eluding to diagnosis and management. In V4, 5 responders insinuated an intrusion diagnosis, and in V2, 11 responders suggested they were confused whether the luxated permanent tooth was a primary or permanent tooth. This uncertainty was also raised responses to V1 and V3. Half of the responders were aware of dental services which could treat dental trauma and minority of responders eluded to the need for follow-up.

### Access

When asked if the ED was an appropriate environment for paediatric dental trauma to present, almost half (n=45) thought that it is appropriate; while n=16 disagreed, and n=33 were unsure.

A common theme raised was the need to improve dental teaching amongst the paediatric emergency medical team, leading some responders to conclude that the PED is not an appropriate place for a dental trauma to present. Proposed improvements included an easy-to-use decision-making tool, regular training of staff including online resources, and suggestions that dental trauma should be included in the medical undergraduate curriculum.

"Doctors have almost no dental training as part of their course and there is little teaching available currently. My decision above would change if we had teaching and a decision-making tool…"

Many responders commented how access depends on the configuration of local services. They noted that ED attendance would be appropriate if there was a structured urgent referral service for dental trauma, including improved communication with dental colleagues. Other suggestions to improve management included establishing emergency dental networks and ‘more streamlined community dental services, in particular staff urgent and emergency dentists with professionals who are comfortable in dealing with children.’

Numerous responders concluded that the PED was the most appropriate place because of a ‘deficit of community dental provision…’ to treat paediatric dental trauma. One responder added: ‘It is not the best place for management but often the only option for patients—most dental trauma presents at weekends or out of hours’. There was acknowledgement that dental hospitals can provide complex emergency dental treatment in working hours but paediatric dental services were limited out of hours.

Responders commented that if the injury was localised to the teeth, it would be more appropriate to attend a GDP, not the PED. However, many responders noted that children are often referred to ED by GDPs. Some responders recognised that management of injuries, such as avulsion requiring reimplantation, is time critical and therefore a PED should be able to manage this and it is appropriate.

"Depends on the mechanism of injury and any concurrent (non-dental) injuries. For isolated dental trauma, GDP is far more appropriate."

It was suggested that more information and resources should be available to signpost parents where to take their child if they have dental trauma because ‘not many parents are aware they can take children to the dental hospital, and therefore, wait a long time in ED before they are referred to the dental or max fax team.’ It was suggested that resources should include ‘clear information for families (in multiple languages) about home first aid for dental trauma, and the most appropriate resources within the region’.

## Discussion

This study provides insight into ED health professionals’ experience, perceptions, awareness and confidence in managing TDIs specifically within a PED setting, addressing both primary and secondary objectives.

TDIs are more common than childhood asthma, yet it is often overlooked in medical and nursing curriculums, as well as within ED education programmes. It is widely reported that medical clinicians’ knowledge of TDI management is lacking, this study demonstrates how an overwhelming number of responders identified the need for more TDI teaching, with only 12 responders reporting that they received some form of teaching on dental trauma as part of their undergraduate studies, comparable to the findings from studies undertaken in various countries.^12–19 22^

Responders’ awareness of how to recognise and manage TDIs was markedly varied. It is noted that TDIs are not life threatening, however, the results of a dental trauma can cause undue psychological affects throughout childhood, in addition they can potentially cause undesirable dental consequences. Therefore, it is important for all ED clinicians to have an awareness of TDI recognition and management. There was an overall lack of awareness of the need of TDI follow-up, which to the authors knowledge, has not been reported in previous published studies. Parents should be made aware of the potential complications and the importance of regular recalls with their dentist. Furthermore, there was little awareness of need for splinting luxation injuries, taking radiographs and a minority of responders displayed awareness of potential non-accidental injuries associated with TDIs. Hence, there is scope to improve the dental teaching received as part of the UK undergraduate curriculum in medicine and within nursing training programme and other associated healthcare training. This supports recent proposals to develop an online dental trauma course for medical professionals.^23 24^

A common theme raised by responders was confusion whether the injured teeth in the vignettes were primary or permanent teeth. The age stated in the vignette question could have informed the clinician whether it was permanent tooth, supporting findings that ED health professionals’ lack knowledge of tooth eruption ages.^22 25^ Presentation and management of TDIs in children is notably different to adults. Paediatric TDIs may involve immature anterior permanent teeth and cooperation can vary. Thus, understanding eruption ages of permanent teeth is of great significance. There is an argument that depending on the specialty, medical clinicians may not require dental trauma knowledge. However, dental trauma could readily present to EDs, GPs, it can be caused by intubation by anaesthetic teams, and could happen to any patient under any medical specialty.

ED health professionals’ confidence in assessing TDIs was notably low, comparable to a previous study.^26^ However, this study assessed all health professionals who may encounter dental trauma in the PED, not just paediatric emergency physicians, finding that seniority is not directly associated with confidence and knowledge, opposing the results from another study.^25^

Many health professionals raised concerns that there is a lack of emergency dental services in their regions within the UK, resulting in many families choosing to attend a PED, echoing findings of a previous study.^25^ The two major children’s units within this study both have OMFS oncall and demonstrated an element of dependency towards oncall OMFS staff. However, many district hospitals do not have this configuration and require an onwards referal to larger regional units with an oncall dentally qualified clinician. This may not only be inconvenient for the child and their family, but also increases the time between injury and management, impacting the prognosis of the tooth.

Responders suggested improving signposting for families and the need for a universal TDI decision-making tool. This could be used by all dental and non-dental health professionals to identify the urgency of the TDI and direct children to the best place for management. In order to improve the current services in the UK, more research is required to understand the use of available services. Managed clinical networks could help advance the services available.^27^

It had been acknowledged that there are limitations to this study. Overall response rate was 63% which is not uncommon for a research survey. However, a higher response rate would have been beneficial. Despite this, the cohort of responders represented a range of ED health professionals who appropriately addressed the aim. Response bias cannot be excluded; those more interested, competent or confident in management of TDIs are more likely to respond to the survey. In order to be more generalisable, it would be preferential to extend the survey to other PEDs in the UK but it should be noted that AHH and BCH cover a large proportion of the North West and Midlands, England, and the staff have been trained in a variety of units across the country.

## Conclusions

Health professionals within two large regional PEDs in the UK recognise that there is a need to enhance dental trauma teaching for ED clinicians who regularly encounter TDIs, to increase their confidence and enable them to triage and advise families appropriately.

An improved pathway between dental and emergency services within these regions of the UK is required for TDI patients and it would be beneficial to improve sign posting for families, and create a decision-making tool for non-dental clinicians to ensure children with a TDI receive the required management and follow-up. This could, in turn, improve outcomes and experience for children who experience a TDI.

Further research should be undertaken to help improve the emergency management of TDIs in children within the UK.

## Supplementary Material

Reviewer comments

Author's manuscript

## Data Availability

Data are available on reasonable request.
