# Supplementary material for: Management of traumatic dental injuries: a survey of paediatric emergency department health professionals
Source: BMJ Paediatr Open. 2023 Mar 22;7(1):e001740. doi: 10.1136/bmjpo-2022-001740 (PMC10040074; doi:10.1136/bmjpo-2022-001740)
Supplement: Author's manuscript [file bmjpo-2022-001740.draft_revisions.pdf]

BMJ Paediatrics Open

BMJ Paediatrics Open is committed to open peer review. As part of this commitment we make the peer review history of every article we publish publicly available.

When an article is published we post the peer reviewers' comments and the authors' responses online. We also post the versions of the paper that were used during peer review. These are the versions that the peer review comments apply to.

The versions of the paper that follow are the versions that were submitted during the peer review process. They are not the versions of record or the final published versions. They should not be cited or distributed as the published version of this manuscript.

BMJ Paediatrics Open is an open access journal and the full, final, typeset and author-corrected version of record of the manuscript is available on our site with no access controls, subscription charges or pay-per-view fees (http://bmjpaedsopen.bmj.com).

If you have any questions on BMJ Paediatrics Open's open peer review process please email info.bmjpo@bmj.com

BMJ Paediatrics Open

Paediatric Emergency Departments clinicians' experience, confidence and awareness of traumatic dental injuries

Journal:	*BMJ Paediatrics Open*
Manuscript ID	bmjpo-2022-001740
Article Type:	Original research
Date Submitted by the Author:	31-Oct-2022
Complete List of Authors:	Gallichan, Nathalie; University of Liverpool; Alder Hey Children's NHS Foundation Trust<br>Albadri, Sondos; University of Liverpool School of Dentistry, Paediatric Department; Alder Hey Children's NHS Foundation Trust<br>Watkins, Francine; University of Liverpool Faculty of Health and Life Sciences<br>Jarad, Fadi; University of Liverpool School of Dentistry, Restorative Department<br>Messahel, Shrouk; Alder Hey Children's NHS Foundation Trust<br>Hartshorn, Stuart; Birmingham Children's Hospital, Emergency Department; University of Birmingham, Birmingham Clinical Trials Unit<br>Gartshore, Laura; University of Liverpool School of Dentistry, Paediatric Dentistry Department
Keywords:	Dentistry, Data Collection

SCHOLARONE™
Manuscripts

I, the Submitting Author has the right to grant and does grant on behalf of all authors of the Work (as defined in the below author licence), an exclusive licence and/or a non-exclusive licence for contributions from authors who are: i) UK Crown employees; ii) where BMJ has agreed a CC-BY licence shall apply, and/or iii) in accordance with the terms applicable for US Federal Government officers or employees acting as part of their official duties; on a worldwide, perpetual, irrevocable, royalty-free basis to BMJ Publishing Group Ltd ("BMJ") its licensees and where the relevant Journal is co-owned by BMJ to the co-owners of the Journal, to publish the Work in this journal and any other BMJ products and to exploit all rights, as set out in our licence.

The Submitting Author accepts and understands that any supply made under these terms is made by BMJ to the Submitting Author unless you are acting as an employee on behalf of your employer or a postgraduate student of an affiliated institution which is paying any applicable article publishing charge ("APC") for Open Access articles. Where the Submitting Author wishes to make the Work available on an Open Access basis (and intends to pay the relevant APC), the terms of reuse of such Open Access shall be governed by a Creative Commons licence – details of these licences and which Creative Commons licence will apply to this Work are set out in our licence referred to above.

Other than as permitted in any relevant BMJ Author's Self Archiving Policies, I confirm this Work has not been accepted for publication elsewhere, is not being considered for publication elsewhere and does not duplicate material already published. I confirm all authors consent to publication of this Work and authorise the granting of this licence.

Confidential: For Review Only

TITLE PAGE

Title

Paediatric Emergency Departments clinicians' experience, confidence and awareness of traumatic dental injuries

Confidential: For Review Only

Corresponding author:

Dr Nathalie Gallichan
The Green, Back Lane, Duddon, Tarporley, Cheshire, England, CW6 0EY
ngalli@liverpool.ac.uk
Tel: (0044) 7747 598 916

Co-authors:
Professor Sondos Albadri
School of Dentistry, University of Liverpool, and Alder Hey Children's NHS Foundation Trust
Liverpool, UK

Professor Francine Watkins
Institute of Population Health, University of Liverpool
Liverpool, UK

Professor Fadi Jarad
School of Dentistry, University of Liverpool
Liverpool, UK

Dr Shrouk Messahel:
Emergency Department, Alder Hey Children's NHS Foundation Trust
Liverpool UK

Dr Stuart Hartshorn
Birmingham Women's and Children's Hospital NHS Foundation Trust and Birmingham Clinical Trials Unit, University of Birmingham
Birmingham, UK

Dr Laura Gartshore
School of Dentistry, University of Liverpool
Liverpool, UK

MeSH terms: Dentists; tooth injuries; child; surveys and questionnaires; emergency service, hospital.

Word count: 2460

Abstract

Objective: To assess Paediatric Emergency Department (PED) clinicians' confidence, experience and awareness in managing traumatic dental injuries (TDIs).

Design: A cross sectional online questionnaire.

Setting: Paediatric Emergency Departments at Alder Hey Children's Hospital and Birmingham Children's Hospital.

Results: 94 clinicians responded. Almost a third of responders (28%, n=26) encounter children with dental trauma daily or weekly. TDI teaching during undergraduate training was received by 13% (n=12) of responders, and 32% (n=30) had never received training. Responders thought they would benefit from online resources and regular teaching on paediatric TDIs, in addition to an easy-to-use decision-making tool to signpost families.

Clinicians' confidence in giving advice to families following a TDI, and in recognising types of TDIs, was notably low; -79 and -76 Net Promotor Score (NPS) respectively.

Responders' awareness of how to recognise and manage TDIs was varied. Majority were aware of the need to attempt to reimplant an avulsed permanent tooth, and the need to refer a child presenting with a complex permanent tooth injury to the oncall dentist. However, very few responders commented on the importance of follow up. Responders also raised concerns about the lack of dental services to treat TDIs in children.

Conclusions There is a need to enhance dental trauma teaching for all emergency department clinicians who encounter TDIs to increase their confidence and enable them to triage and advise patients appropriately. Additionally, increased signposting for families to the appropriate service could in turn improve outcomes and experience for children who experience a TDI.

Confidential: For Review Only

INTRODUCTION

Paediatric Emergency Departments (PEDs) are increasingly being utilised as providers of dental care within the UK. Dental presentations predominantly include those attending with dental caries or traumatic dental injuries (TDIs).[1 2] It is estimated that 40% of children experience a TDI to their primary teeth and up to a third of children sustain trauma to their permanent teeth.[3-6] If TDIs were included in the world's most frequent diseases, it could rank fifth.[7]

TDIs can usually be acutely managed by a general dental practitioner (GDP), however, families may choose to attend Emergency Departments (EDs) where they are triaged and assessed by ED clinicians. The optimal management of TDIs in the ED relies on a child being appropriately triaged, seeking advice from the oncall dentist if required, and appropriate advice being given. Some ED units may not have an oncall dentist. Inappropriate management and advice may lead to undesirable consequences including damage to developing permanent tooth, pulpal necrosis leading to pain and dental abscess, or resorption and ultimate loss of a permanent tooth.[8]

The provision of oral health education in undergraduate medical programmes is reported to be minimal, and TDI management is often non-existant.[9 10] However, ED doctors are required to have knowledge of dental trauma as part of their specialty training. [11] Studies have reported that medical clinicians' knowledge of TDI management is lacking. Most studies focused on avulsion management and many are not generalisable to the UK national health service's provision of emergency care.[12-19] To the authors' knowledge, this is the first reported study to of ED clinicians' confidence and knowledge of TDIs in a paediatric emergency setting within the UK.

The study was designed to assess Paediatric Emergency Department (PED) clinician experience, confidence, and awareness of TDIs. Primary objectives were to determine clinician confidence in assessing and managing TDIs in children. Secondary objectives were to identify the training and education clinicians have received, to identify PED clinician awareness of resources which aid TDI management, and investigate how they manage TDIs.

Confidential: For Review Only

METHOD

Study design, sample and distribution

The study was undertaken as a cross sectional survey, distributed November 2020. Two of the largest PEDs in the UK were invited to participate: Alder Hey Children's Hospital (AHH) and Birmingham Children's Hospital (BCH). AHH cares for 330,000 children in the North West of England each year. BCH cares for 270,600 children in the West Midlands each year.

ED clinicians who encounter TDIs were invited to participate; including ED doctors, nurses, and nurse practitioners. The target sample size was 150 clinicians across the two PEDs.

Participation was voluntary and opt-in. A pre-notification email was sent to clinicians one week in advance to brief the target population of the study, via a PED Consultant identified at each unit. A reminder email was sent two weeks later. Four weeks after the initial email, the survey closed.

Questionnaire design and content

A self-administered online questionnaire was designed to gather quantitative and qualitative data. The anonymous questionnaire was constructed using Microsoft Forms and informed by literature review. The questionnaire was pre-tested for content and face validity amongst medical and dental professionals, a group of experts in TDI management, and a methodological expert. Written and verbal feedback was encouraged.

Responder demographics and experience in dental trauma management was collected and analysed using descriptive statistics. Open and closed questions, Likert scales and theoretical clinical vignettes were designed to assess TDI awareness, responders' confidence and pragmatic decision-making. Qualitative answers were analysed and grouped into recurring answers and also into themes. The STROBE cross sectional checklist was used when writing the report.[20]

Confidential: For Review Only

RESULTS

Clinicians' roles and experience

Of 94 responders, the majority were nursing staff (n=39, 42%), followed by trainee doctors (n=24, 26%) and ED consultants (n=21, 22%) (table 1). 64.9% identified themselves as working in the North West and 35.1% from the West Midlands respectively. Experience of working in PED was relatively evenly split; <1 year (n=23), 1-5 years (n=23), 5-10 years (n=21) and >10 years (n=27). Majority of responders encountered children with dental trauma a few-times-a-month (n=51, 54.3%), 24.5% (n=23) weekly, 18.1% (n=17) a few-times-a-year, and 3.2% (n=3) encountered it daily.

TABLE 1: CLINICIAN ROLE WITHIN THE PED	COUNT
**ADVANCED NURSE/CLINICAL PRACTITIONER (ANP/ACP)**	11
**ADVANCED NURSE PRACTITIONER TRAINEE**	1
**CONSULTANT**	21
**EMERGENCY NURSE PRACTITIONER**	7
**HEALTH CARE ASSISTANT**	1
**NURSE**	23
**PHYSICIAN ASSOCIATE**	2
**SPECIALTY DOCTOR/ ASSOCIATE SPECIALIST**	4
**TRAINEE DOCTOR/ CLINICAL FELLOW (MIDDLE GRADE/ REGISTRAR)**	12
**TRAINEE DOCTOR/CLINICAL FELLOW (JUNIOR LEVEL- CORE TRAINEE/SHO EQUIVALENT/GP TRAINEE)**	12
GRAND TOTAL	**94**

Teaching and education received on dental trauma

A third of responders, 32% (n=30) never received teaching about TDIs. 19% (n=18) received teaching during post-graduate training, and 13% (n=12) during undergraduate training (figure 1). The majority, 51% (n=48), reported training 'on the job, whilst working in the PED'. Figure 2 demonstrates the format of the teaching received.

The majority of responders, 67% (n=63) reported they never used a TDI decision-making-pathway in their department; 20% (n=19) sometimes used one, and 13% (n=12) regularly used one. Those who never used a TDI decision-making-pathway had lower confidence scores than those who did. A third

Confidential: For Review Only

of responders used resources including the dental trauma guide website,[21] 'internet', teaching notes, or an ED handbook to aid TDI recognition.

Confidence

When providing advice to families following a TDI, and in recognising types of TDIs, responder's confidence was notably low; -79 and -76 Net Promotor Score (NPS) respectively (figure 3). 77-80% of responders stated their confidence was less than 6/10 (figure 4). When sub-analysed, consultants had the most confidence, followed by ANPs, with lowest confidence amongst physician associates. Trainee doctors and middle grades had notably lower confidence compared to ED consultants.

Awareness of dental trauma and vignette responses

Thematic analysis of the responders comments to the vignettes (V1-4), including figures 5a-d, led to the identification of the following themes:

- OMFS referral and utilisation for TDIs
- Mixed awareness diagnosing and management of TDIs
- Lack of confidence in managing TDIs
- Confusion over recognising primary or permanent teeth
- Limited follow up advise provided

When confronted with a complex intrusion and fracture injury in a permanent tooth (V4), 77% (n=72) of responders would refer to OMFS, compared to 23% (n=22) referring for a primary tooth luxation injury (V3). V3 also elicited some responders (n=7, 7.4%) to refer to plastics or ENT.

Overall, 85 responders (90.4%) would advise to reimplant the avulsed tooth (V1) or place the tooth in an appropriate medium. In V1 and V2, 10 responders (10.6%) mentioned splinting the injured tooth, in V4, no responders mentioned splinting. Responders highlighted a lack of confidence in reimplanting a tooth, commenting *'…Would not feel confident in placing tooth in situ also not fully sure if it is appropriate.'* Two responders would not provide any management for V1 except reassurance.

Confidential: For Review Only

There was awareness from some responders that V3 was an intruded primary tooth (12, 12.8%) and some also displayed awareness that it '*may have impacted or damaged the permanent tooth'.* V2 and V4 were more complex TDIs, with less responders eluding to diagnosis and management. In V4, 5.3% (n=5) insinuated an intrusion diagnosis, and in V2 11.7% (n=11) suggested they were confused whether the luxated permanent tooth was a primary or permanent tooth. This uncertainty was also raised responses to V1 and V3. Half of the responders were aware of dental services which could treat dental trauma and minority of responders eluded to the need for follow up.

Access

When asked if the ED was an appropriate environment for paediatric dental trauma to present, 48% (n=45) thought that is appropriate; whilst 17% (n=16) disagreed, and 35% (n=33) were unsure.

A common theme raised was the need to improve dental teaching amounst the medical team, leading some responders to conclude that the PED is not an appropriate place for a dental trauma to present. Proposed improvements included an easy-to-use decision-making tool, regular training of staff including online resources, and suggestions that dental trauma should be included in the medical undergraduate curriculum.

> *'Doctors have almost no dental training as part of their course and there is little teaching available currently. My decision above would change if we had teaching and a decision-making tool…'*

Many responders commented how access depends on the configuration of local services. They noted that ED attendance would be appropriate if there was a structured urgent referral service for dental trauma, including improved communication with dental colleagues. Other suggestions to improve management included establishing emergency dental networks and *'more streamlined community*

Confidential: For Review Only

dental services, in particular staff urgent and emergency dentists with professionals who are comfortable in dealing with children.'

Numerous responders concluded that the PED was the most appropriate place because of a '*deficit of community dental provision…'* to treat paediatric dental trauma. One responder added: *'It is not the best place for management but often the only option for patients- most dental trauma presents at weekends or out of hours'.* There was acknowledgment that dental hospitals can provide complex emergency dental treatment in working hours but paediatric dental services were limited out of hours.

Responders commented that if the injury was localised to the teeth, it would be more appropriate to attend a GDP, not the PED. However, many responders noted that children are often referred to ED by GDPs. Some responders recognised that management of injuries, such as avulsion requiring re-implantation, is time critical and therefore a PED should be able to manage this and it is appropriate.

> *'Depends on the mechanism of injury and any concurrent (non-dental) injuries. For isolated dental trauma, GDP is far more appropriate.'*

It was suggested that more information and resouces should be available to signpost parents where to take their child if they have dental trauma because '*not many parents are aware they can take children to the dental hospital and therefore wait a long time in ED before they are referred to the dental or max fax team.'* It was suggested that resources should include *'clear information for families (in multiple languages) about home first aid for dental trauma, and the most appropriate resources within the region'.*

DISCUSSION

This study provides insight into emergency department clinicians' experience, perceptions, awareness and confidence in managing TDIs specifically within a Paediatric Emergency Department setting, addressing both primary and secondary objectives.

Confidential: For Review Only

TDIs are more common than childhood asthma, yet it is often overlooked in both medical curriculums and ED education programmes. It is widely reported that medical clinicians' knowledge of TDI management is lacking, this study demonstrates how an overwhelming number of responders identified the need for more TDI teaching, with only 13% of responders reporting that they received some form of teaching on dental trauma as part of their undergraduate studies, comparable to the findings from studies undertaken in various countries. [12-19 22]

Responders' awareness of how to recognise and manage TDIs was markedly varied. It is noted that TDIs are not life threatening, however, the results of a dental trauma can cause undue psychological affects throughout childhood, in addition they can potentially cause undesirable dental consequences. Therefore, it is important for all ED clinicians to have an awareness of TDI recognition and management. There was an overall lack of awareness of the need of TDI follow up, which to the authors knowledge, has not been reported in previous published studies. Parents should be made aware of the potential complications and the importance of regular recalls with their dentist. Furthermore, there was little awareness of need for splinting luxation injuries, taking radiographs, and a minority of responders displayed awareness of potential non-accidental injuries (NAI) associated with TDIs. Hence, there is scope to improve the dental teaching received as part of the UK undergraduate curriculum, supporting recent proposals to develop an online dental trauma course for medical professionals. [23 24]

A common theme raised by responders, was confusion whether the injured teeth in the vignettes were primary or permanent teeth. The age stated in the vignette question could have informed the clinician whether it was permanent tooth, supporting findings that medical clinicians' lack knowledge of eruption ages.[22 25] Presentation and management of TDIs in children is notably different to adults. Paediatric TDIs may involve immature anterior permanent teeth, and cooperation can vary. Thus, understanding eruption ages of permanent teeth is of great significance. There is an argument that depending on the specialty, medical clinicians may not require dental trauma knowledge. However,

Confidential: For Review Only

dental trauma could readily present to EDs, GPs, it can be caused by intubation by anaesthetic teams, and could happen to any patient under any medical specialty.

Clinicians confidence in assessing TDIs was notably low, comparable to a previous study.[26] However, this study assessed all clinicians who may encounter dental trauma in the PED, not just paediatric emergency physicians, finding that seniority is not directly associated with confidence and knowledge, opposing the results from another study.[25]

Many clinicians raised concerns that there is a lack of emergency dental services in the UK, resulting in many families choosing to attend a PED, echoing findings of a previous study.[25] The two major children's units within this study both have OMFS oncall and demonstrated an element of dependency towards oncall OMFS staff. However, many district hospitals do not have this configuration and require an onwards referal to larger regional units with an oncall dentally qualified clinician. This may not only be inconvenient for the child and their family, but also increases the time between injury and management, impacting the prognosis of the tooth.

Responders suggested improving signposting for families and the need for a universal TDI decision-making-tool. This could be utilised by all dental and non-dental clinicians to identify the urgency of the TDI and direct children to the best place for management. In order to improve the current services in the UK, more research is required to understand the use of available services. Managed clinical networks (MCNs) could help advance the services available.

It had been acknowledged that there are limitations to this study. Overall response rate was 63% which is not uncommon for a research survey. However, a higher response rate would have been more beenficial. Despite this, the cohort of responders represented a range of clinicians who appropriately addressed the aim. Response bias cannot be excluded; those more interested, competent, or confident in management of TDIs are more likely to respond to the survey. Arguably, more PEDs across the UK could be included, however, this study represents two large areas of the UK, therefore the

Confidential: For Review Only

results are generalisable in the UK, and are relatable to other countries where children attend EDs with TDIs.

CONCLUSIONS

Medical clinicians recognise that there is a need to enhance dental trauma teaching for emergency department clinicians who regularly encounter TDIs, to increase their confidence and enable them to triage and advise families appropriately.

An improved pathway between dental and emergency services is required for TDI patients and it would be beneficial to improve sign posting for families, and create a decision-making tool for non-dental clinicians to ensure children with a TDI receive the required management and follow up. This could, in turn, improve outcomes and experience for children who experience a TDI.

Further research should be undertaken to help improve the emergency management of TDIs in children within the UK.

ETHICS STATEMENT

Ethical approval was obtained by the Health and Life Sciences Research Ethics Committee, University of Liverpool, reference number 7585.

COMPETING INTEREST

None declared

ACKNOWLEDGMENTS

Thank you to the staff at Alder Hey Children's Hospital and Birmingham Children's Hospital Emergency Departments who frequently manage dental patients and kindly participated in this study.

Confidential: For Review Only

FUNDING STATEMENT

This research received no specific grant from any funding agency in the public, commercial or not-for-profit sectors.

CONTRIBUTIONS

N Gallichan: Conceptualisation, methodology, investigation, data curation, writing- original draft preparation. **S Albadri**: Conceptualisation, methodology, writing- reviewing and editing, visualisation and supervision. **F Watkins**: Methodology, writing- reviewing and editing, investigation. **F Jarad:** Methodology, supervision. **S Messahel**: Conceptualisation, project administration, writing- reviewing and editing. **S Hartshorn**: Project administration, writing- reviewing and editing. **L Gartshore:** Conceptualisation, methodology, supervision**,** writing- reviewing and editing, visualisation

STROBE 2007 (v4) Statement—Checklist of items that should be included in reports of *cross-sectional studies*

Section/Topic	Item #	Recommendation	Reported on page #
**Title and abstract**	1	(*a*) Indicate the study's design with a commonly used term in the title or the abstract	1
		(*b*) Provide in the abstract an informative and balanced summary of what was done and what was found	2
**Introduction**			
Background/rationale	2	Explain the scientific background and rationale for the investigation being reported	3
Objectives	3	State specific objectives, including any prespecified hypotheses	3
**Methods**			
Study design	4	Present key elements of study design early in the paper	4
Setting	5	Describe the setting, locations, and relevant dates, including periods of recruitment, exposure, follow-up, and data collection	4
Participants	6	(*a*) Give the eligibility criteria, and the sources and methods of selection of participants	4

Confidential: For Review Only

Variables	7	Clearly define all outcomes, exposures, predictors, potential confounders, and effect modifiers. Give diagnostic criteria, if applicable	4
Data sources/ measurement	8*	For each variable of interest, give sources of data and details of methods of assessment (measurement). Describe comparability of assessment methods if there is more than one group	4
Bias	9	Describe any efforts to address potential sources of bias	4
Study size	10	Explain how the study size was arrived at	4
Quantitative variables	11	Explain how quantitative variables were handled in the analyses. If applicable, describe which groupings were chosen and why	4
Statistical methods	12	(*a*) Describe all statistical methods, including those used to control for confounding	4
		(*b*) Describe any methods used to examine subgroups and interactions	4
		(*c*) Explain how missing data were addressed	n/a
		(*d*) If applicable, describe analytical methods taking account of sampling strategy	4
		(*e*) Describe any sensitivity analyses	n/a
**Results**			
Participants	13*	(a) Report numbers of individuals at each stage of study—eg numbers potentially eligible, examined for eligibility, confirmed eligible, included in the study, completing follow-up, and analysed	5
		(b) Give reasons for non-participation at each stage	n/a
		(c) Consider use of a flow diagram	n/a
Descriptive data	14*	(a) Give characteristics of study participants (eg demographic, clinical, social) and information on exposures and potential confounders	5
		(b) Indicate number of participants with missing data for each variable of interest	n/a
Outcome data	15*	Report numbers of outcome events or summary measures	5
Main results	16	(*a*) Give unadjusted estimates and, if applicable, confounder-adjusted estimates and their precision (eg, 95% confidence interval). Make clear which confounders were adjusted for and why they were included	5
		(*b*) Report category boundaries when continuous variables were categorized	5
		(*c*) If relevant, consider translating estimates of relative risk into absolute risk for a meaningful time period	n/a
Other analyses	17	Report other analyses done—eg analyses of subgroups and interactions, and sensitivity analyses	5
**Discussion**			
Key results	18	Summarise key results with reference to study objectives	8

Confidential: For Review Only

Limitations	19	Discuss limitations of the study, taking into account sources of potential bias or imprecision. Discuss both direction and magnitude of any potential bias	10
Interpretation	20	Give a cautious overall interpretation of results considering objectives, limitations, multiplicity of analyses, results from similar studies, and other relevant evidence	10
Generalisability	21	Discuss the generalisability (external validity) of the study results	10
**Other information**			
Funding	22	Give the source of funding and the role of the funders for the present study and, if applicable, for the original study on which the present article is based	n/a-12

DATA SHARING

Data is available on request

What is already known on this topic

- There is a lack of dental teaching within undergraduate medical programmes, and often no teaching on dental trauma despite how commonly presents to medical clinicians.
- Timely acute management of dental trauma has a direct impact on the prognosis of an injured tooth.
- TDIs should be followed up by a dentist as per dental trauma guidelines.

What this study adds

- There is a demand to develop an easy-to-use decision-making tool for non-dental clinicians, ensuring children with TDIs receive the required management and follow up.
- There is a need to sign post families towards the most efficient and effective place to help manage a TDI.
- Clinicians working in Paediatric Emergency Departments would benefit from online resources and regular teaching on paediatric TDIs.

REFERENCES

1. Heggie C, Gallichan N, Gartshore L, et al. Traumatic and non-traumatic dental presentations to the Paediatric Emergency Departments of two UK Children's Hospitals: a multi-centre evaluation. *International Journal of Paediatric Dentistry* 2021

Confidential: For Review Only

2. Marshman Z, Broomhead T, Rodd HD, et al. Who attends a Children's Hospital Emergency Department for dental reasons? A two-step cluster analysis approach. *Community Dent Oral Epidemiol* 2017;45(1):49-58. doi: 10.1111/cdoe.12258 [published Online First: 2016/09/30]
3. Andreasen J, Ravn J. Epidemiology of traumatic dental injuries to primary and permanent teeth in a Danish population sample. *International journal of oral surgery* 1972;1(5):235-39.
4. Hamilton FA, Hill FJ, Mackie IC. Investigation of lay knowledge of the management of avulsed permanent incisors. Denmark: MUNKSGAARD - SUBSCRIPTION SERVICE, 1997:19.
5. Chadwick BL, White D, Morris A, et al. Non-carious tooth conditions in children in the UK, 2003. *British dental journal* 2006;200(7):379.
6. O'Brien M. Children's dental health in the United Kingdom 1993: HMSO 1994.
7. Petti S, Glendor U, Andersson L. World traumatic dental injury prevalence and incidence, a meta-analysis-One billion living people have had traumatic dental injuries. *Dental Traumatology* 2018;34(2):71-86. doi: 10.1111/edt.12389
8. Welbury R, Duggal MS, Hosey MT. Paediatric dentistry: Oxford university press 2018.
9. Abbott B, Zybutz C, Scott KM, et al. A review of the hours dedicated to oral health education in medical programmes across Australia. *Internal Medicine Journal* 2018;48(9):1035-40. doi: 10.1111/imj.14021
10. Aren A, Erdem AP, Aren G, et al. Importance of knowledge of the management of traumatic dental injuries in emergency departments. *Ulus Travma Acil Cerrahi Derg* 2018;24(2):136-44.
11. RCEM C. Paediatric Emergency Medicine, Sub-specialty Syllabus.
12. Abu-Dawoud M, Al-Enezi B, Andersson L. Knowledge of emergency management of avulsed teeth among young physicians and dentists. *Dental Traumatology* 2007;23(6):348-55.
13. Bahammam LA. Knowledge and attitude of emergency physician about the emergency management of tooth avulsion. *BMC oral health* 2018;18(1):1-9.
14. Holan G, Shmueli Y. Knowledge of physicians in hospital emergency rooms in Israel on their role in cases of avulsion of permanent incisors. *International Journal of Paediatric Dentistry* 2003;13(1):13-19.
15. Ulusoy AT, ÖNDER H, Cetin B, et al. Knowledge of medical hospital emergency physicians about the first-aid management of traumatic tooth avulsion. *International journal of paediatric dentistry* 2012;22(3):211-16.
16. Yigit Y, Helvacioglu-Yigit D, Kan B, et al. Dentofacial traumatic injuries: A survey of knowledge and attitudes among emergency medicine physicians in Turkey. *Dental Traumatology* 2019;35(1):20-26.
17. Subhashraj K. Awareness of management of dental trauma among medical professionals in Pondicherry, India. *Dental Traumatology* 2009;25(1):92-94.
18. Zencircioglu OK, Eden E, Ocek ZA. Access to health care after dental trauma in children: A quantitative and qualitative evaluation, 2019:163-70.
19. Trivedy C, Kodate N, Ross A, et al. The attitudes and awareness of emergency department (ED) physicians towards the management of common dentofacial emergencies. *Dental Traumatology* 2012;28(2):121-26.
20. Von Elm E, Altman DG, Egger M, et al. The Strengthening the Reporting of Observational Studies in Epidemiology (STROBE) statement: guidelines for reporting observational studies. *Bulletin of the World Health Organization* 2007;85:867-72.
21. Copenhagen RCfRODaDoOaM-FSatUHo. Dental Trauma Guide Website: Resource Centre for Rare Oral Diseases and Department of Oral and Maxillo-Facial Surgery at the University Hospital of Copenhagen; 2021 [Available from: https://dentaltraumaguide.org/2021.
22. Patel K, Driscoll P. Dental knowledge of accident and emergency senior house officers. *Emergency Medicine Journal* 2002;19(6):539-41.
23. Yeng T, O'Sullivan AJ, Shulruf B. A Proposal to Introduce Dental Trauma into Medical Education: An Insight. *Dental Traumatology* 2020

Confidential: For Review Only

24. Yeng T, O'Sullivan AJ, Shulruf B. Online dental trauma course for medical education. *Dental Traumatology* 2021

25. Samaei H, Weiland TJ, Dilley S, et al. Knowledge and confidence of a convenience sample of Australasian emergency doctors in managing dental emergencies: results of a survey. *Emergency medicine international* 2015;2015

26. Cully M, Cully J, Nietert PJ, et al. Physician Confidence in Dental Trauma Treatment and the Introduction of a Dental Trauma Decision-Making Pathway for the Pediatric Emergency Department. *Pediatric Emergency Care* 2019;35(11):745-48. doi: 10.1097/PEC.0000000000001479

Confidential: For Review Only

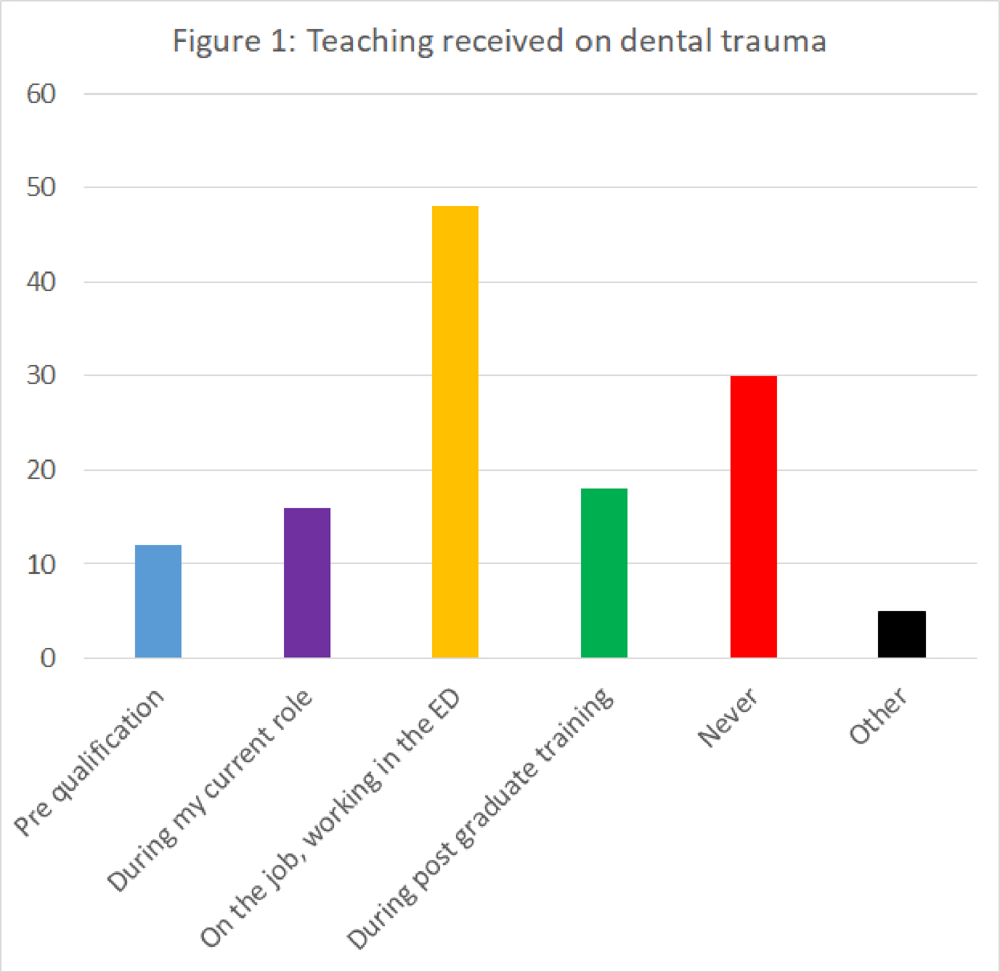

Figure 1: Teaching on dental trauma

109x106mm (144 x 144 DPI)

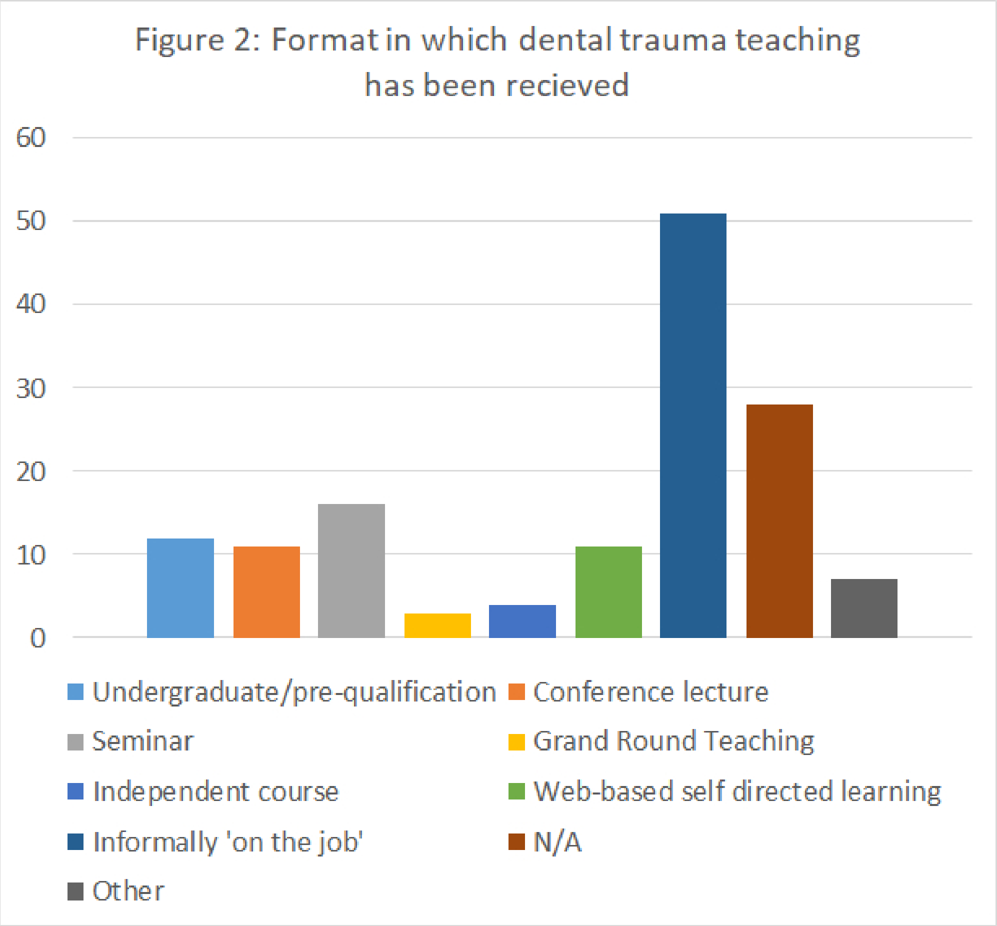

Figure 2: Format in which dental trauma teaching has been received

108x100mm (144 x 144 DPI)

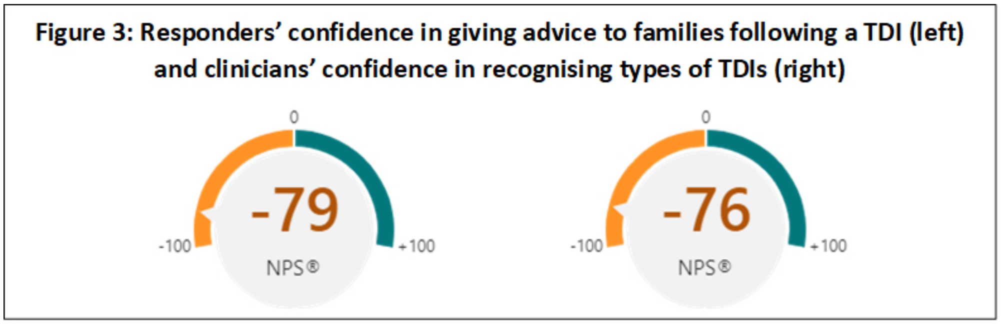

Figure 3: Responders confidence

139x44mm (144 x 144 DPI)

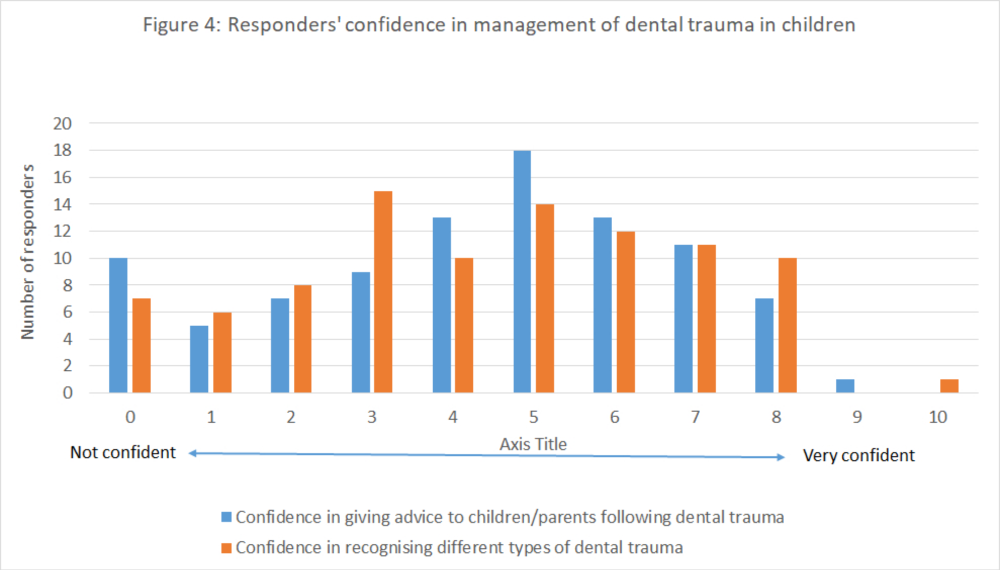

Figure 4: responders confidence graph

174x99mm (144 x 144 DPI)

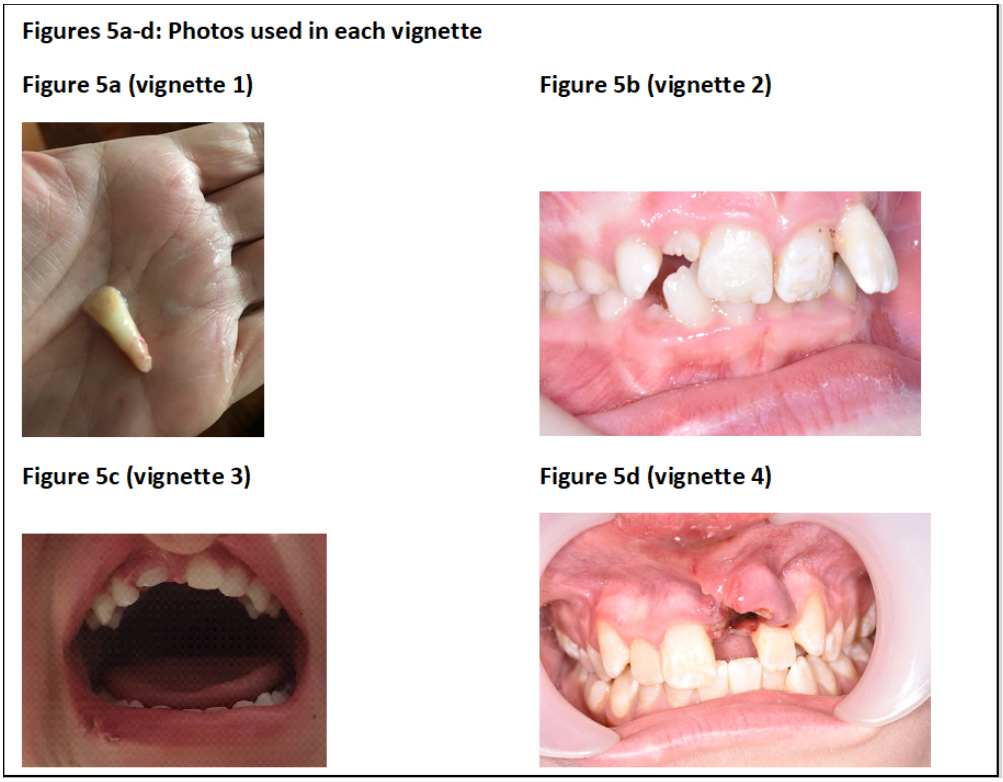

Figure 5: Vignette photos 1-4

160x124mm (144 x 144 DPI)

BMJ Paediatrics Open

Management of traumatic dental injuries: a survey of Paediatric Emergency Department health professionals

Journal:	*BMJ Paediatrics Open*
Manuscript ID	bmjpo-2022-001740.R1
Article Type:	Original research
Date Submitted by the Author:	19-Dec-2022

Complete List of Authors:	Gallichan, Nathalie; University of Liverpool; Alder Hey Children's NHS Foundation Trust<br>Albadri, Sondos; University of Liverpool School of Dentistry, Paediatric Department; Alder Hey Children's NHS Foundation Trust<br>Watkins, Francine; University of Liverpool Faculty of Health and Life Sciences<br>Jarad, Fadi; University of Liverpool School of Dentistry, Restorative Department<br>Messahel, Shrouk; Alder Hey Children's NHS Foundation Trust<br>Hartshorn, Stuart; Birmingham Children's Hospital, Emergency Department; University of Birmingham, Birmingham Clinical Trials Unit<br>Gartshore, Laura; University of Liverpool School of Dentistry, Paediatric Dentistry Department

Keywords:	Dentistry, Data Collection

SCHOLARONE™
Manuscripts

I, the Submitting Author has the right to grant and does grant on behalf of all authors of the Work (as defined in the below author licence), an exclusive licence and/or a non-exclusive licence for contributions from authors who are: i) UK Crown employees; ii) where BMJ has agreed a CC-BY licence shall apply, and/or iii) in accordance with the terms applicable for US Federal Government officers or employees acting as part of their official duties; on a worldwide, perpetual, irrevocable, royalty-free basis to BMJ Publishing Group Ltd ("BMJ") its licensees and where the relevant Journal is co-owned by BMJ to the co-owners of the Journal, to publish the Work in this journal and any other BMJ products and to exploit all rights, as set out in our licence.

The Submitting Author accepts and understands that any supply made under these terms is made by BMJ to the Submitting Author unless you are acting as an employee on behalf of your employer or a postgraduate student of an affiliated institution which is paying any applicable article publishing charge ("APC") for Open Access articles. Where the Submitting Author wishes to make the Work available on an Open Access basis (and intends to pay the relevant APC), the terms of reuse of such Open Access shall be governed by a Creative Commons licence – details of these licences and which Creative Commons licence will apply to this Work are set out in our licence referred to above.

Other than as permitted in any relevant BMJ Author's Self Archiving Policies, I confirm this Work has not been accepted for publication elsewhere, is not being considered for publication elsewhere and does not duplicate material already published. I confirm all authors consent to publication of this Work and authorise the granting of this licence.

Confidential: For Review Only

TITLE PAGE

Title

Management of traumatic dental injuries: a survey of Paediatric Emergency Department health professionals

Corresponding author:

Dr Nathalie Gallichan
The Green, Back Lane, Duddon, Tarporley, Cheshire, England, CW6 0EY
ngalli@liverpool.ac.uk
Tel: (0044) 7747 598 916

Co-authors:
Professor Sondos Albadri
School of Dentistry, University of Liverpool, and Alder Hey Children's NHS Foundation Trust
Liverpool, UK

Professor Francine Watkins
Institute of Population Health, University of Liverpool
Liverpool, UK

Professor Fadi Jarad
School of Dentistry, University of Liverpool
Liverpool, UK

Dr Shrouk Messahel:
Emergency Department, Alder Hey Children's NHS Foundation Trust
Liverpool UK

Dr Stuart Hartshorn
Birmingham Women's and Children's Hospital NHS Foundation Trust and Birmingham Clinical Trials Unit, University of Birmingham
Birmingham, UK

Dr Laura Gartshore
School of Dentistry, University of Liverpool
Liverpool, UK

MeSH terms: Dentists; tooth injuries; child; surveys and questionnaires; emergency service, hospital.

Word count: 2460

Confidential: For Review Only

Abstract

Objective: To assess Paediatric Emergency Department (PED) health professionals' confidence, experience and awareness in managing traumatic dental injuries (TDIs).

Design: A cross sectional online survey.

Setting: Paediatric Emergency Departments at Alder Hey Children's Hospital and Birmingham Children's Hospital.

Results: 94 ED health professionals responded. A third of responders (n=26) encounter children with dental trauma daily or weekly. TDI teaching during undergraduate training was received by 13% (n=12) of responders, and 32% (n=30) had never received training. Responders thought they would benefit from online resources and regular teaching on paediatric TDIs, in addition to an easy-to-use decision-making tool to signpost families.

ED health professionals' confidence in giving advice to families following a TDI, and in recognising types of TDIs, was notably low; -79 and -76 Net Promotor Score (NPS) respectively.

Responders' awareness of how to recognise and manage TDIs was varied. Majority were aware of the need to attempt to reimplant an avulsed permanent tooth, and the need to refer a child presenting with a complex permanent tooth injury to the oncall dentist. However, very few responders commented on the importance of follow up. Responders also raised concerns about the lack of dental services to treat TDIs in children.

Conclusions There is a need to enhance dental trauma teaching for all emergency department health professionals who encounter TDIs to increase their confidence and enable them to triage and advise patients appropriately. Additionally, increased signposting for families to the appropriate service could in turn improve outcomes and experience for children who experience a TDI.

Confidential: For Review Only

What is already known on this topic

- There is a lack of dental teaching within undergraduate medical programmes, and often no teaching on dental trauma despite how commonly presents to medical health professionals.
- Timely acute management of dental trauma has a direct impact on the prognosis of an injured tooth.

What this study adds

- There is a demand to develop an easy-to-use decision-making tool for health professionals working in a PED
- There is a need to signpost families towards the most efficient and effective place to help manage a TDI.
- Health professionals working in Paediatric Emergency Departments would benefit from online resources and regular teaching on paediatric TDIs.

How this study might affect research, practice or policy

- Initiate discussions within regional MCNs regarding paediatric acute trauma pathways.
- Creation of a formal teaching element and resources on paediatric dental trauma for health professionals working in PEDs.
- Encourage further research across the UK into paediatric acute trauma pathways.

Confidential: For Review Only

INTRODUCTION

Paediatric Emergency Departments (PEDs) are increasingly being utilised as providers of dental care within the UK. [1 2] Dental presentations predominantly include those attending with dental caries or traumatic dental injuries (TDIs). It is estimated that 40% of children experience a TDI to their primary teeth and up to a third of children sustain trauma to their permanent teeth.[3-6] If TDIs were included in the world's most frequent diseases, it could rank fifth.[7]

TDIs can usually be acutely managed by a general dental practitioner (GDP), however, families may choose to attend Emergency Departments (EDs) where they are triaged and assessed by ED health professionals. The optimal management of TDIs in the ED relies on a child being appropriately triaged, seeking advice from the oncall dentist if required, and appropriate advice being given. Some ED units may not have an oncall dentist. Inappropriate management and advice may lead to undesirable consequences including damage to developing permanent tooth, pulpal necrosis leading to pain and dental abscess, or resorption and ultimate loss of a permanent tooth.[8]

The provision of oral health education in undergraduate medical programmes is reported to be minimal, and TDI management is often non-existant.[9 10] However, ED doctors are required to have knowledge of dental trauma as part of their specialty training. [11] Studies have reported that medical health professionals' knowledge of TDI management is lacking. Most studies focused on avulsion management and many are not generalisable to the UK national health service's provision of emergency care.[12-19] The study was designed to assess Paediatric Emergency Department (PED) clinician experience, confidence, and awareness of TDIs. Primary objectives were to determine clinician confidence in assessing and managing TDIs in children. Secondary objectives were to identify the training and education clinicians have received, to identify PED clinician awareness of resources which aid TDI management, and investigate how they manage TDIs.

Confidential: For Review Only

METHOD

Study design, sample and distribution

The study was undertaken as a cross sectional survey, distributed November 2020 using convenience sampling, inviting all ED health professionals working in two of the largest PEDs to participate: Alder Hey Children's Hospital (AHH) and Birmingham Children's Hospital (BCH). AHH cares for 330,000 children in the North West of England and North Wales each year. BCH cares for 270,600 children in the West Midlands and mid Wales each year.

The target sample size was total number of health professionals who worked across the two PEDs (n=150). All members of staff who may triage, provide advice or manage children with a TDI were included and invited to participate, including ED doctors, nurses, and nurse practitioners. There were no exclusions.

Participation was voluntary and opt-in. A pre-notification email was sent to health professionals one week in advance to brief the target population of the study, via a PED Consultant identified at each unit. A reminder email was sent two weeks later. Four weeks after the initial email, the survey closed.

Survey design and content

A self-administered online questionnaire was designed to gather quantitative and qualitative data, a copy can be found in the appendices. The anonymous questionnaire was constructed using Microsoft Forms and informed by literature review. All responses were confidential, untracked and IP addresses were not stored. The survey has been pre-tested for content and face validity using face-face feedback discussion with a paediatric dentist clinician and researcher and subsequently piloted among a convenience sample of clinicians who have experience working in ED to further inform development. Written and verbal feedback was encouraged and time to complete the questionnaire was recorded.

Responder demographics and experience in dental trauma management was collected and analysed using descriptive statistics. A total of 21 questions were asked including open and closed questions,

Confidential: For Review Only

Likert scales and theoretical clinical vignettes, designed to assess TDI awareness, responders' confidence and pragmatic decision-making. Analysis was undertaken using Microsoft Forms and Microsoft Excel (2019). Confidence was assessed using a Likert scale and presented using a Net Promotor Score (NPS) which provides a number from -100 to 100 calculated by subtracting the percentage of detractors from the percentage of promoters, and excludes the passive responses. Qualitative answers were analysed and grouped into recurring answers and also into themes. All questions were compulsory in order to complete and submit the survey, therefore there was no missing data. The CROSS checklist was used when writing the report.[20]

RESULTS

ED health professionals' roles and experience

Of 94 responders, the majority were doctors (n= 49), and 45 responders were nursing staff. (table 1). 64.9% identified themselves as working in the North West and 35.1% from the West Midlands respectively. Experience of working in PED was relatively evenly split; <1 year (n=23), 1-5 years (n=23), 5-10 years (n=21) and >10 years (n=27). Majority of responders encountered children with dental trauma a few-times-a-month (n=51), 23 responders encounter TDIs weekly, 3 responders encounter TDIs in children daily, and 17 responders encounter TDIs a few times a year. There was no missing data all questions were required to complete the questionnaire. Overall response rate was 63%.

https://mc.manuscriptcentral.com/bmjpo

Confidential: For Review Only

TABLE 1: CLINICIAN ROLE WITHIN THE PED	COUNT
**ADVANCED NURSE/CLINICAL PRACTITIONER (ANP/ACP)**	11
**ADVANCED NURSE PRACTITIONER TRAINEE**	1
**CONSULTANT**	21
**EMERGENCY NURSE PRACTITIONER**	7
**HEALTH CARE ASSISTANT**	1
**NURSE**	23
**PHYSICIAN ASSOCIATE**	2
**SPECIALTY DOCTOR/ ASSOCIATE SPECIALIST**	4
**TRAINEE DOCTOR/ CLINICAL FELLOW (MIDDLE GRADE/ REGISTRAR)**	12
**TRAINEE DOCTOR/CLINICAL FELLOW (JUNIOR LEVEL- CORE TRAINEE/SHO EQUIVALENT/GP TRAINEE)**	12
GRAND TOTAL	**94**

Teaching and education received on dental trauma

A third of responders, (n=30) never received teaching about TDIs. 18 received teaching during post-graduate training, and n=12 during undergraduate training (figure 1). The majority, (n=48), reported training 'on the job, whilst working in the PED'. Figure 2 demonstrates the format of the teaching received.

The majority of responders, (n=63) reported they never used a TDI decision-making-pathway in their department; 19 responders sometimes used one, and 12 responders regularly used one. Those who never used a TDI decision-making-pathway had lower confidence scores than those who did. A third of responders used resources including the dental trauma guide website,[21] 'internet', teaching notes, or an ED handbook to aid TDI recognition.

Confidence

When providing advice to families following a TDI, and in recognising types of TDIs, responder's confidence was notably low; -79 and -76NPS respectively. 77-80% of responders stated their confidence was less than 6/10 (figure 3). When sub-analysed, consultants had the most confidence, followed by ANPs, with lowest confidence amongst physician associates. Trainee doctors and middle grades had notably lower confidence compared to ED consultants.

https://mc.manuscriptcentral.com/bmjpo

Confidential: For Review Only

Awareness of dental trauma and vignette responses

Thematic analysis of the responders comments to the vignettes (V1-4), including figures 4a-d, led to the identification of the following themes:

- Oral Maxillofacial Surgery (OMFS) referral and utilisation for TDIs
- Mixed awareness diagnosing and management of TDIs
- Lack of confidence in managing TDIs
- Confusion over recognising primary or permanent teeth
- Limited follow up advise provided

When confronted with a complex intrusion and fracture injury in a permanent tooth (V4), 72responders would refer to OMFS, compared to 22 who would refer for a primary tooth luxation injury (V3). V3 also elicited some responders (n=7) to refer to plastics or ENT.

Overall, 85 responders (90.4%) would advise to reimplant the avulsed tooth (V1) or place the tooth in an appropriate medium. In V1 and V2, 10 responders (10.6%) mentioned splinting the injured tooth, in V4, no responders mentioned splinting. Responders highlighted a lack of confidence in reimplanting a tooth, commenting *'…Would not feel confident in placing tooth in situ also not fully sure if it is appropriate.'* Two responders would not provide any management for V1 except reassurance.

There was awareness from some responders that V3 was an intruded primary tooth (n=12) and some also displayed awareness that it *'may have impacted or damaged the permanent tooth'.* V2 and V4 were more complex TDIs, with less responders eluding to diagnosis and management. In V4, 5 responders insinuated an intrusion diagnosis, and in V2 11 responders suggested they were confused whether the luxated permanent tooth was a primary or permanent tooth. This uncertainty was also raised responses to V1 and V3. Half of the responders were aware of dental services which could treat dental trauma and minority of responders eluded to the need for follow up.

Confidential: For Review Only

Access

When asked if the ED was an appropriate environment for paediatric dental trauma to present, almost half(n=45) thought that is appropriate; whilst n=16 disagreed, and n=33 were unsure.

A common theme raised was the need to improve dental teaching amounst the paediatric emergency medical team, leading some responders to conclude that the PED is not an appropriate place for a dental trauma to present. Proposed improvements included an easy-to-use decision-making tool, regular training of staff including online resources, and suggestions that dental trauma should be included in the medical undergraduate curriculum.

> *'Doctors have almost no dental training as part of their course and there is little teaching available currently. My decision above would change if we had teaching and a decision-making tool…'*

Many responders commented how access depends on the configuration of local services. They noted that ED attendance would be appropriate if there was a structured urgent referral service for dental trauma, including improved communication with dental colleagues. Other suggestions to improve management included establishing emergency dental networks and *'more streamlined community dental services, in particular staff urgent and emergency dentists with professionals who are comfortable in dealing with children.'*

Numerous responders concluded that the PED was the most appropriate place because of a *'deficit of community dental provision…'* to treat paediatric dental trauma. One responder added: *'It is not the best place for management but often the only option for patients- most dental trauma presents at weekends or out of hours'.* There was acknowledgment that dental hospitals can provide complex emergency dental treatment in working hours but paediatric dental services were limited out of hours.

Confidential: For Review Only

Responders commented that if the injury was localised to the teeth, it would be more appropriate to attend a GDP, not the PED. However, many responders noted that children are often referred to ED by GDPs. Some responders recognised that management of injuries, such as avulsion requiring re-implantation, is time critical and therefore a PED should be able to manage this and it is appropriate.

> *'Depends on the mechanism of injury and any concurrent (non-dental) injuries. For isolated dental trauma, GDP is far more appropriate.'*

It was suggested that more information and resouces should be available to signpost parents where to take their child if they have dental trauma because '*not many parents are aware they can take children to the dental hospital and therefore wait a long time in ED before they are referred to the dental or max fax team.'* It was suggested that resources should include *'clear information for families (in multiple languages) about home first aid for dental trauma, and the most appropriate resources within the region'.*

DISCUSSION

This study provides insight into emergency department health professionals' experience, perceptions, awareness and confidence in managing TDIs specifically within a Paediatric Emergency Department setting, addressing both primary and secondary objectives.

TDIs are more common than childhood asthma, yet it is often overlooked in medical and nursing curriculums, as well as within ED education programmes. It is widely reported that medical clinicians' knowledge of TDI management is lacking, this study demonstrates how an overwhelming number of responders identified the need for more TDI teaching, with only 12 responders reporting that they received some form of teaching on dental trauma as part of their undergraduate studies, comparable to the findings from studies undertaken in various countries. [12-19 22]

Responders' awareness of how to recognise and manage TDIs was markedly varied. It is noted that TDIs are not life threatening, however, the results of a dental trauma can cause undue psychological

https://mc.manuscriptcentral.com/bmjpo

Confidential: For Review Only

affects throughout childhood, in addition they can potentially cause undesirable dental consequences. Therefore, it is important for all ED clinicians to have an awareness of TDI recognition and management. There was an overall lack of awareness of the need of TDI follow up, which to the authors knowledge, has not been reported in previous published studies. Parents should be made aware of the potential complications and the importance of regular recalls with their dentist. Furthermore, there was little awareness of need for splinting luxation injuries, taking radiographs, and a minority of responders displayed awareness of potential non-accidental injuries (NAI) associated with TDIs. Hence, there is scope to improve the dental teaching received as part of the UK undergraduate curriculum in medicine and within nursing training programme and other associated healthcare training. This supports recent proposals to develop an online dental trauma course for medical professionals. [23 24]

A common theme raised by responders, was confusion whether the injured teeth in the vignettes were primary or permanent teeth. The age stated in the vignette question could have informed the clinician whether it was permanent tooth, supporting findings that ED health professionals' lack knowledge of tooth eruption ages.[22 25] Presentation and management of TDIs in children is notably different to adults. Paediatric TDIs may involve immature anterior permanent teeth, and cooperation can vary. Thus, understanding eruption ages of permanent teeth is of great significance. There is an argument that depending on the specialty, medical clinicians may not require dental trauma knowledge. However, dental trauma could readily present to EDs, GPs, it can be caused by intubation by anaesthetic teams, and could happen to any patient under any medical specialty.

ED health professionals' confidence in assessing TDIs was notably low, comparable to a previous study.[26] However, this study assessed all health professionals who may encounter dental trauma in the PED, not just paediatric emergency physicians, finding that seniority is not directly associated with confidence and knowledge, opposing the results from another study.[25]

https://mc.manuscriptcentral.com/bmjpo

Confidential: For Review Only

Many health professionals raised concerns that there is a lack of emergency dental services in their regions within the UK, resulting in many families choosing to attend a PED, echoing findings of a previous study.[25] The two major children's units within this study both have OMFS oncall and demonstrated an element of dependency towards oncall OMFS staff. However, many district hospitals do not have this configuration and require an onwards referal to larger regional units with an oncall dentally qualified clinician. This may not only be inconvenient for the child and their family, but also increases the time between injury and management, impacting the prognosis of the tooth.

Responders suggested improving signposting for families and the need for a universal TDI decision-making-tool. This could be utilised by all dental and non-dental health professionals to identify the urgency of the TDI and direct children to the best place for management. In order to improve the current services in the UK, more research is required to understand the use of available services. Managed clinical networks (MCNs) could help advance the services available.[27]

It had been acknowledged that there are limitations to this study. Overall response rate was 63% which is not uncommon for a research survey. However, a higher response rate would have been beneficial. Despite this, the cohort of responders represented a range of ED health professionals who appropriately addressed the aim. Response bias cannot be excluded; those more interested, competent, or confident in management of TDIs are more likely to respond to the survey. In order to be more generalisable, it would be preferential to extend the survey to other PEDs in the UK but it should be noted that AHH and BCH cover a large proportion of the North West and Midlands, England, and the staff have been trained in a variety of units across the country.

CONCLUSIONS

Confidential: For Review Only

Health professionals within two large regional PEDs in the UK recognise that there is a need to enhance dental trauma teaching for emergency department clinicians who regularly encounter TDIs, to increase their confidence and enable them to triage and advise families appropriately.

An improved pathway between dental and emergency services within these regions of the UK is required for TDI patients and it would be beneficial to improve sign posting for families, and create a decision-making tool for non-dental clinicians to ensure children with a TDI receive the required management and follow up. This could, in turn, improve outcomes and experience for children who experience a TDI.

Further research should be undertaken to help improve the emergency management of TDIs in children within the UK.

ETHICS STATEMENT

Ethical approval was obtained by the Health and Life Sciences Research Ethics Committee, University of Liverpool, reference number 7585.

Patient and public involvement statement

There was no patient or public involvement in this study

COMPETING INTEREST

None declared

ACKNOWLEDGMENTS

Thank you to the staff at Alder Hey Children's Hospital and Birmingham Children's Hospital Emergency Departments who frequently manage dental patients and kindly participated in this study.

FUNDING STATEMENT

Confidential: For Review Only

This research received no specific grant from any funding agency in the public, commercial or not-for-profit sectors.

CONTRIBUTIONS

N Gallichan: Conceptualisation, methodology, investigation, data curation, writing- original draft preparation. **S Albadri**: Conceptualisation, methodology, writing- reviewing and editing, visualisation and supervision. **F Watkins**: Methodology, writing- reviewing and editing, investigation. **F Jarad:** Methodology, supervision. **S Messahel**: Conceptualisation, project administration, writing- reviewing and editing. **S Hartshorn**: Project administration, writing- reviewing and editing. **L Gartshore:** Conceptualisation, methodology, supervision**,** writing- reviewing and editing, visualisation

DATA SHARING

Data is available on request

REFERENCES

1. Heggie C, Gallichan N, Gartshore L, et al. Traumatic and non-traumatic dental presentations to the Paediatric Emergency Departments of two UK Children's Hospitals: a multi-centre evaluation. *International Journal of Paediatric Dentistry* 2021;32(1):90-100.2. Marshman Z, Broomhead T, Rodd HD, et al. Who attends a Children's Hospital Emergency Department for dental reasons? A two-step cluster analysis approach. *Community Dent Oral Epidemiol* 2017;45(1):49-58. doi: 10.1111/cdoe.12258 [published Online First: 2016/09/30]
3. Andreasen J, Ravn J. Epidemiology of traumatic dental injuries to primary and permanent teeth in a Danish population sample. *International journal of oral surgery* 1972;1(5):235-39.
4. Hamilton FA, Hill FJ, Mackie IC. Investigation of lay knowledge of the management of avulsed permanent incisors. *Dental Traumatology* .1997;13(1):19-23..
Hamilton FA, Hill FJ, Mackie IC. Investigation of lay knowledge of the management of avulsed permanent incisors.. 1997 Feb
5. Chadwick BL, White D, Morris A, et al. Non-carious tooth conditions in children in the UK, 2003. *British Dental Journal* 2006;200(7):379.
6. O'Brien M. Children's dental health in the United Kingdom 1993: HM Stationery Office; 1994.7. Petti S, Glendor U, Andersson L. World traumatic dental injury prevalence and incidence, a meta-analysis-One billion living people have had traumatic dental injuries. *Dental Traumatology* 2018;34(2):71-86. doi: 10.1111/edt.12389
8. Welbury R, Duggal MS, Hosey MT. Paediatric dentistry: Oxford university press 2018.
9. Abbott B, Zybutz C, Scott KM, et al. A review of the hours dedicated to oral health education in medical programmes across Australia. *Internal Medicine Journal* 2018;48(9):1035-40. doi: 10.1111/imj.14021

https://mc.manuscriptcentral.com/bmjpo

Confidential: For Review Only

10. Aren A, Erdem AP, Aren G, et al. Importance of knowledge of the management of traumatic dental injuries in emergency departments. *Ulus Travma Acil Cerrahi Derg* 2018;24(2):136-44.
11. RCEM C. Paediatric Emergency Medicine, Sub-specialty Syllabus. [online] 2021. https://www.rcpch.ac.uk/sites/default/files/2021-09/Appendix%204M%20-%20Paediatric%20Emergency%20Medicine.pdf (accessed 3rd November 2021)
12. Abu-Dawoud M, Al-Enezi B, Andersson L. Knowledge of emergency management of avulsed teeth among young physicians and dentists. *Dental Traumatology* 2007;23(6):348-55.
13. Bahammam LA. Knowledge and attitude of emergency physician about the emergency management of tooth avulsion. *BMC oral health* 2018;18(1):1-9.
14. Holan G, Shmueli Y. Knowledge of physicians in hospital emergency rooms in Israel on their role in cases of avulsion of permanent incisors. *International Journal of Paediatric Dentistry* 2003;13(1):13-19.
15. Ulusoy AT, ÖNDER H, Cetin B, et al. Knowledge of medical hospital emergency physicians about the first-aid management of traumatic tooth avulsion. *International journal of paediatric dentistry* 2012;22(3):211-16.
16. Yigit Y, Helvacioglu-Yigit D, Kan B, et al. Dentofacial traumatic injuries: A survey of knowledge and attitudes among emergency medicine physicians in Turkey. *Dental Traumatology* 2019;35(1):20-26.
17. Subhashraj K. Awareness of management of dental trauma among medical professionals in Pondicherry, India. *Dental Traumatology* 2009;25(1):92-94.
18. Zencircioglu OK, Eden E, Ocek ZA. Access to health care after dental trauma in children: A quantitative and qualitative evaluation, 2019:163-70.
19. Trivedy C, Kodate N, Ross A, et al. The attitudes and awareness of emergency department (ED) physicians towards the management of common dentofacial emergencies. *Dental Traumatology* 2012;28(2):121-26.
20. Sharma A, Minh Duc NT, Luu Lam Thang T, et al. A consensus-based checklist for reporting of survey studies (CROSS). *Journal of general internal medicine* 2021;36(10):3179-87.
21. Dental Trauma Guide Website: Resource Centre for Rare Oral Diseases and Department of Oral and Maxillo-Facial Surgery at the University Hospital of Copenhagen; [online] 2021. https://dentaltraumaguide.org/. (accessed 13th October 2022)
22. Patel K, Driscoll P. Dental knowledge of accident and emergency senior house officers. *Emergency Medicine Journal* 2002;19(6):539-41.
23. Yeng T, O'Sullivan AJ, Shulruf B. A Proposal to Introduce Dental Trauma into Medical Education: An Insight. *Dental Traumatology* 2020; 36(4):390-2.24. Yeng T, O'Sullivan AJ, Shulruf B. Online dental trauma course for medical education. *Dental Traumatology.* 2021;37(6):803-6.25. Samaei H, Weiland TJ, Dilley S, et al. Knowledge and confidence of a convenience sample of Australasian emergency doctors in managing dental emergencies: results of a survey. *Emergency medicine international* 2015;4:2015.
26. Cully M, Cully J, Nietert PJ, et al. Physician Confidence in Dental Trauma Treatment and the Introduction of a Dental Trauma Decision-Making Pathway for the Pediatric Emergency Department. *Pediatric Emergency Care* 2019;35(11):745-48. doi: 10.1097/PEC.0000000000001479
27. NHS England. Commissioning Standard for Dental Specialties – Paediatric Dentistry. Leeds. [online] 2018. https://www.england.nhs.uk/wp-content/uploads/2018/04/commissioning-standard-for-dental-specialties-paediatric-dentristry.pdf (accessed 6th December 2022).

Confidential: For Review Only

Confidential: For Review Only

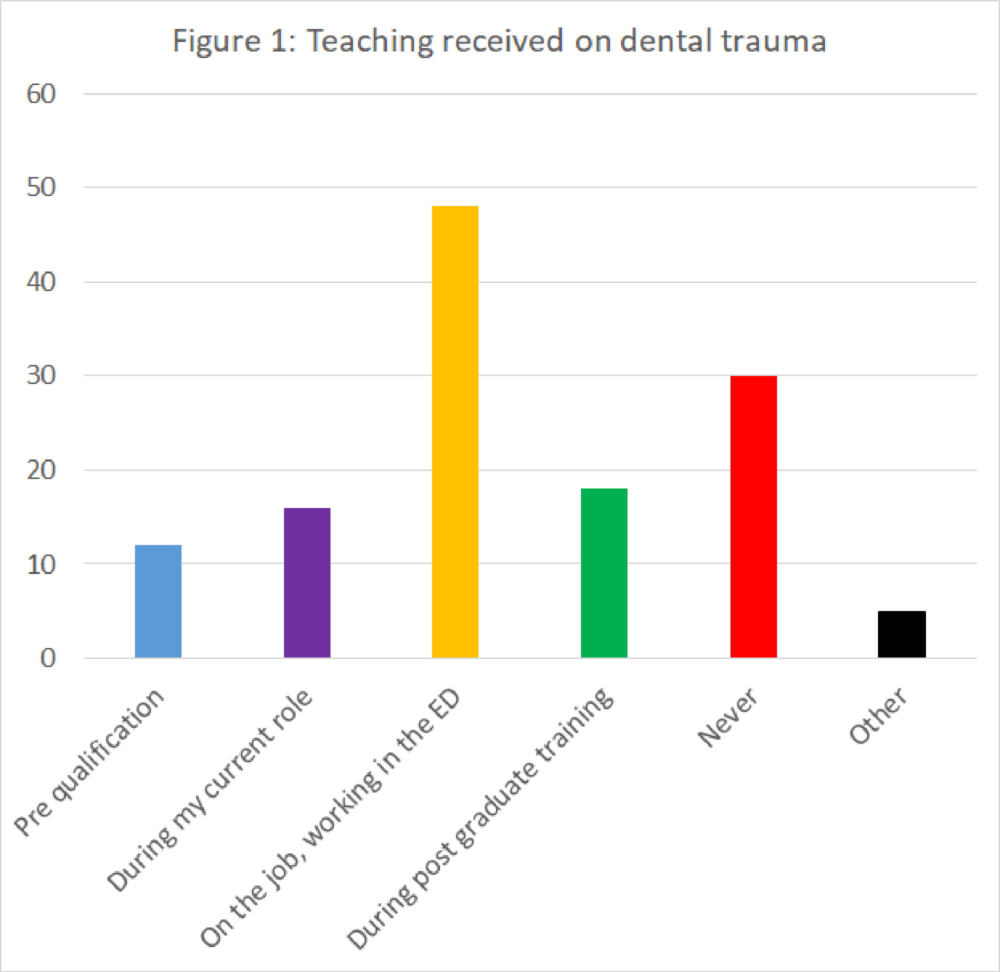

Figure 1: Teaching on dental trauma

109x106mm (144 x 144 DPI)

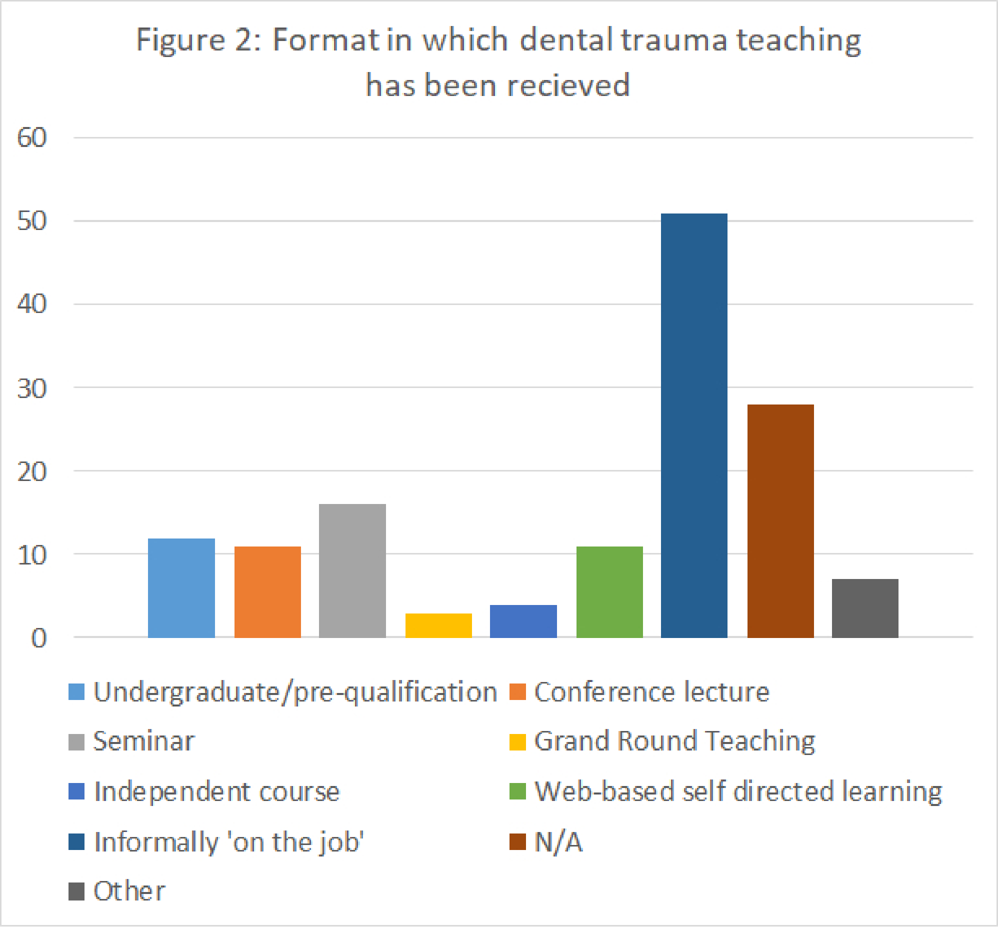

Figure 2: Format in which dental trauma teaching has been received

108x100mm (144 x 144 DPI)

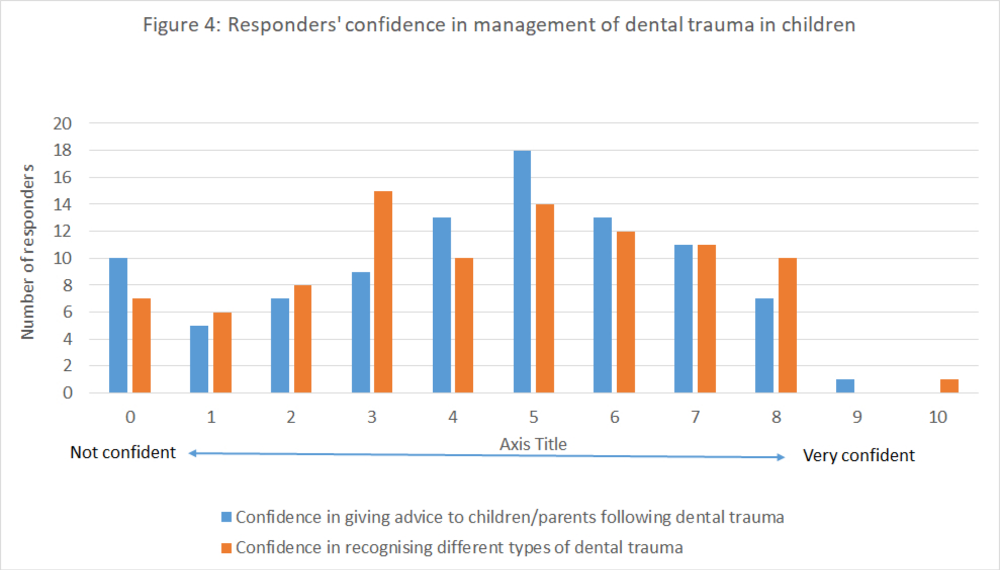

Figure 3: responders confidence

174x99mm (144 x 144 DPI)

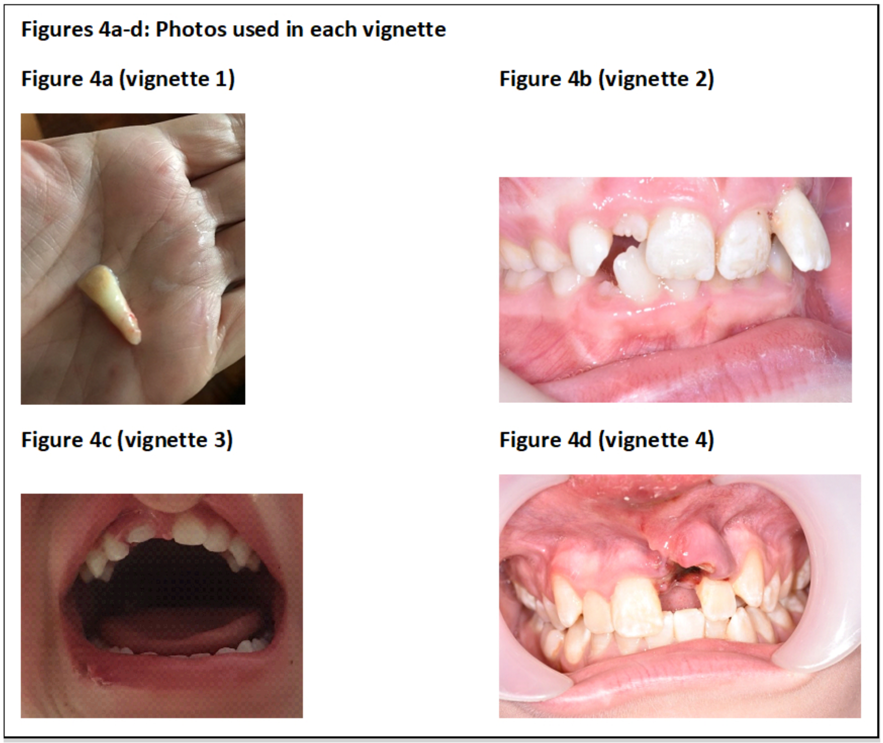

Figures 4a-d: Photos used in vignettes 1-4

174x132mm (144 x 144 DPI)

BMJ Paediatrics Open

Management of traumatic dental injuries: a survey of Paediatric Emergency Department health professionals

Journal:	*BMJ Paediatrics Open*
Manuscript ID	bmjpo-2022-001740.R1
Article Type:	Original research
Date Submitted by the Author:	19-Dec-2022

Complete List of Authors:	Gallichan, Nathalie; University of Liverpool; Alder Hey Children's NHS Foundation Trust<br>Albadri, Sondos; University of Liverpool School of Dentistry, Paediatric Department; Alder Hey Children's NHS Foundation Trust<br>Watkins, Francine; University of Liverpool Faculty of Health and Life Sciences<br>Jarad, Fadi; University of Liverpool School of Dentistry, Restorative Department<br>Messahel, Shrouk; Alder Hey Children's NHS Foundation Trust<br>Hartshorn, Stuart; Birmingham Children's Hospital, Emergency Department; University of Birmingham, Birmingham Clinical Trials Unit<br>Gartshore, Laura; University of Liverpool School of Dentistry, Paediatric Dentistry Department

Keywords:	Dentistry, Data Collection

SCHOLARONE™ Manuscripts

I, the Submitting Author has the right to grant and does grant on behalf of all authors of the Work (as defined in the below author licence), an exclusive licence and/or a non-exclusive licence for contributions from authors who are: i) UK Crown employees; ii) where BMJ has agreed a CC-BY licence shall apply, and/or iii) in accordance with the terms applicable for US Federal Government officers or employees acting as part of their official duties; on a worldwide, perpetual, irrevocable, royalty-free basis to BMJ Publishing Group Ltd ("BMJ") its licensees and where the relevant Journal is co-owned by BMJ to the co-owners of the Journal, to publish the Work in this journal and any other BMJ products and to exploit all rights, as set out in our licence.

The Submitting Author accepts and understands that any supply made under these terms is made by BMJ to the Submitting Author unless you are acting as an employee on behalf of your employer or a postgraduate student of an affiliated institution which is paying any applicable article publishing charge ("APC") for Open Access articles. Where the Submitting Author wishes to make the Work available on an Open Access basis (and intends to pay the relevant APC), the terms of reuse of such Open Access shall be governed by a Creative Commons licence – details of these licences and which Creative Commons licence will apply to this Work are set out in our licence referred to above.

Other than as permitted in any relevant BMJ Author's Self Archiving Policies, I confirm this Work has not been accepted for publication elsewhere, is not being considered for publication elsewhere and does not duplicate material already published. I confirm all authors consent to publication of this Work and authorise the granting of this licence.

Confidential: For Review Only

TITLE PAGE

Title

Management of traumatic dental injuries: a survey of Paediatric Emergency Department health professionals

Corresponding author:

Dr Nathalie Gallichan
The Green, Back Lane, Duddon, Tarporley, Cheshire, England, CW6 0EY
ngalli@liverpool.ac.uk
Tel: (0044) 7747 598 916

Co-authors:
Professor Sondos Albadri
School of Dentistry, University of Liverpool, and Alder Hey Children's NHS Foundation Trust
Liverpool, UK

Professor Francine Watkins
Institute of Population Health, University of Liverpool
Liverpool, UK

Professor Fadi Jarad
School of Dentistry, University of Liverpool
Liverpool, UK

Dr Shrouk Messahel:
Emergency Department, Alder Hey Children's NHS Foundation Trust
Liverpool UK

Dr Stuart Hartshorn
Birmingham Women's and Children's Hospital NHS Foundation Trust and Birmingham Clinical Trials Unit, University of Birmingham
Birmingham, UK

Dr Laura Gartshore
School of Dentistry, University of Liverpool
Liverpool, UK

MeSH terms: Dentists; tooth injuries; child; surveys and questionnaires; emergency service, hospital.

Word count: 2460

Confidential: For Review Only

Abstract

Objective: To assess Paediatric Emergency Department (PED) health professionals' confidence, experience and awareness in managing traumatic dental injuries (TDIs).

Design: A cross sectional online survey.

Setting: Paediatric Emergency Departments at Alder Hey Children's Hospital and Birmingham Children's Hospital.

Results: 94 ED health professionals responded. A third of responders (n=26) encounter children with dental trauma daily or weekly. TDI teaching during undergraduate training was received by 13% (n=12) of responders, and 32% (n=30) had never received training. Responders thought they would benefit from online resources and regular teaching on paediatric TDIs, in addition to an easy-to-use decision-making tool to signpost families.

ED health professionals' confidence in giving advice to families following a TDI, and in recognising types of TDIs, was notably low; -79 and -76 Net Promotor Score (NPS) respectively.

Responders' awareness of how to recognise and manage TDIs was varied. Majority were aware of the need to attempt to reimplant an avulsed permanent tooth, and the need to refer a child presenting with a complex permanent tooth injury to the oncall dentist. However, very few responders commented on the importance of follow up. Responders also raised concerns about the lack of dental services to treat TDIs in children.

Conclusions There is a need to enhance dental trauma teaching for all emergency department health professionals who encounter TDIs to increase their confidence and enable them to triage and advise patients appropriately. Additionally, increased signposting for families to the appropriate service could in turn improve outcomes and experience for children who experience a TDI.

Confidential: For Review Only

What is already known on this topic

- There is a lack of dental teaching within undergraduate medical programmes, and often no teaching on dental trauma despite how commonly presents to medical health professionals.
- Timely acute management of dental trauma has a direct impact on the prognosis of an injured tooth.

What this study adds

- There is a demand to develop an easy-to-use decision-making tool for health professionals working in a PED
- There is a need to signpost families towards the most efficient and effective place to help manage a TDI.
- Health professionals working in Paediatric Emergency Departments would benefit from online resources and regular teaching on paediatric TDIs.

How this study might affect research, practice or policy

- Initiate discussions within regional MCNs regarding paediatric acute trauma pathways.
- Creation of a formal teaching element and resources on paediatric dental trauma for health professionals working in PEDs.
- Encourage further research across the UK into paediatric acute trauma pathways.

Confidential: For Review Only

Confidential: For Review Only

INTRODUCTION

Paediatric Emergency Departments (PEDs) are increasingly being utilised as providers of dental care within the UK. [1 2] Dental presentations predominantly include those attending with dental caries or traumatic dental injuries (TDIs). It is estimated that 40% of children experience a TDI to their primary teeth and up to a third of children sustain trauma to their permanent teeth.[3-6] If TDIs were included in the world's most frequent diseases, it could rank fifth.[7]

TDIs can usually be acutely managed by a general dental practitioner (GDP), however, families may choose to attend Emergency Departments (EDs) where they are triaged and assessed by ED health professionals. The optimal management of TDIs in the ED relies on a child being appropriately triaged, seeking advice from the oncall dentist if required, and appropriate advice being given. Some ED units may not have an oncall dentist. Inappropriate management and advice may lead to undesirable consequences including damage to developing permanent tooth, pulpal necrosis leading to pain and dental abscess, or resorption and ultimate loss of a permanent tooth.[8]

The provision of oral health education in undergraduate medical programmes is reported to be minimal, and TDI management is often non-existant.[9 10] However, ED doctors are required to have knowledge of dental trauma as part of their specialty training. [11] Studies have reported that medical health professionals' knowledge of TDI management is lacking. Most studies focused on avulsion management and many are not generalisable to the UK national health service's provision of emergency care.[12-19] The study was designed to assess Paediatric Emergency Department (PED) clinician experience, confidence, and awareness of TDIs. Primary objectives were to determine clinician confidence in assessing and managing TDIs in children. Secondary objectives were to identify the training and education clinicians have received, to identify PED clinician awareness of resources which aid TDI management, and investigate how they manage TDIs.

METHOD

Study design, sample and distribution

The study was undertaken as a cross sectional survey, distributed November 2020 using convenience sampling, inviting all ED health professionals working in two of the largest PEDs to participate: Alder Hey Children's Hospital (AHH) and Birmingham Children's Hospital (BCH). AHH cares for 330,000 children in the North West of England and North Wales each year. BCH cares for 270,600 children in the West Midlands and mid Wales each year.

The target sample size was total number of health professionals who worked across the two PEDs (n=150). All members of staff who may triage, provide advice or manage children with a TDI were included and invited to participate, including ED doctors, nurses, and nurse practitioners. There were no exclusions.

Participation was voluntary and opt-in. A pre-notification email was sent to health professionals one week in advance to brief the target population of the study, via a PED Consultant identified at each unit. A reminder email was sent two weeks later. Four weeks after the initial email, the survey closed.

Survey design and content

A self-administered online questionnaire was designed to gather quantitative and qualitative data, a copy can be found in the appendices. The anonymous questionnaire was constructed using Microsoft Forms and informed by literature review. All responses were confidential, untracked and IP addresses were not stored. The survey has been pre-tested for content and face validity using face-face feedback discussion with a paediatric dentist clinician and researcher and subsequently piloted among a convenience sample of clinicians who have experience working in ED to further inform development. Written and verbal feedback was encouraged and time to complete the questionnaire was recorded.

Responder demographics and experience in dental trauma management was collected and analysed using descriptive statistics. A total of 21 questions were asked including open and closed questions,

Confidential: For Review Only

Likert scales and theoretical clinical vignettes, designed to assess TDI awareness, responders' confidence and pragmatic decision-making. Analysis was undertaken using Microsoft Forms and Microsoft Excel (2019). Confidence was assessed using a Likert scale and presented using a Net Promotor Score (NPS) which provides a number from -100 to 100 calculated by subtracting the percentage of detractors from the percentage of promoters, and excludes the passive responses. Qualitative answers were analysed and grouped into recurring answers and also into themes. All questions were compulsory in order to complete and submit the survey, therefore there was no missing data. The CROSS checklist was used when writing the report.[20]

RESULTS

ED health professionals' roles and experience

Of 94 responders, the majority were doctors (n= 49), and 45 responders were nursing staff. (table 1). 64.9% identified themselves as working in the North West and 35.1% from the West Midlands respectively. Experience of working in PED was relatively evenly split; <1 year (n=23), 1-5 years (n=23), 5-10 years (n=21) and >10 years (n=27). Majority of responders encountered children with dental trauma a few-times-a-month (n=51), 23 responders encounter TDIs weekly, 3 responders encounter TDIs in children daily, and 17 responders encounter TDIs a few times a year. There was no missing data all questions were required to complete the questionnaire. Overall response rate was 63%.

Confidential: For Review Only

TABLE 1: CLINICIAN ROLE WITHIN THE PED	COUNT
**ADVANCED NURSE/CLINICAL PRACTITIONER (ANP/ACP)**	11
**ADVANCED NURSE PRACTITIONER TRAINEE**	1
**CONSULTANT**	21
**EMERGENCY NURSE PRACTITIONER**	7
**HEALTH CARE ASSISTANT**	1
**NURSE**	23
**PHYSICIAN ASSOCIATE**	2
**SPECIALTY DOCTOR/ ASSOCIATE SPECIALIST**	4
**TRAINEE DOCTOR/ CLINICAL FELLOW (MIDDLE GRADE/ REGISTRAR)**	12
**TRAINEE DOCTOR/CLINICAL FELLOW (JUNIOR LEVEL- CORE TRAINEE/SHO EQUIVALENT/GP TRAINEE)**	12
GRAND TOTAL	**94**

Teaching and education received on dental trauma

A third of responders, (n=30) never received teaching about TDIs. 18 received teaching during post-graduate training, and n=12 during undergraduate training (figure 1). The majority, (n=48), reported training 'on the job, whilst working in the PED'. Figure 2 demonstrates the format of the teaching received.

The majority of responders, (n=63) reported they never used a TDI decision-making-pathway in their department; 19 responders sometimes used one, and 12 responders regularly used one. Those who never used a TDI decision-making-pathway had lower confidence scores than those who did. A third of responders used resources including the dental trauma guide website,[21] 'internet', teaching notes, or an ED handbook to aid TDI recognition.

Confidence

When providing advice to families following a TDI, and in recognising types of TDIs, responder's confidence was notably low; -79 and -76NPS respectively. 77-80% of responders stated their confidence was less than 6/10 (figure 3). When sub-analysed, consultants had the most confidence, followed by ANPs, with lowest confidence amongst physician associates. Trainee doctors and middle grades had notably lower confidence compared to ED consultants.

Confidential: For Review Only

Awareness of dental trauma and vignette responses

Thematic analysis of the responders comments to the vignettes (V1-4), including figures 4a-d, led to the identification of the following themes:

- Oral Maxillofacial Surgery (OMFS) referral and utilisation for TDIs
- Mixed awareness diagnosing and management of TDIs
- Lack of confidence in managing TDIs
- Confusion over recognising primary or permanent teeth
- Limited follow up advise provided

When confronted with a complex intrusion and fracture injury in a permanent tooth (V4), 72responders would refer to OMFS, compared to 22 who would refer for a primary tooth luxation injury (V3). V3 also elicited some responders (n=7) to refer to plastics or ENT.

Overall, 85 responders (90.4%) would advise to reimplant the avulsed tooth (V1) or place the tooth in an appropriate medium. In V1 and V2, 10 responders (10.6%) mentioned splinting the injured tooth, in V4, no responders mentioned splinting. Responders highlighted a lack of confidence in reimplanting a tooth, commenting *'…Would not feel confident in placing tooth in situ also not fully sure if it is appropriate.'* Two responders would not provide any management for V1 except reassurance.

There was awareness from some responders that V3 was an intruded primary tooth (n=12) and some also displayed awareness that it *'may have impacted or damaged the permanent tooth'.* V2 and V4 were more complex TDIs, with less responders eluding to diagnosis and management. In V4, 5 responders insinuated an intrusion diagnosis, and in V2 11 responders suggested they were confused whether the luxated permanent tooth was a primary or permanent tooth. This uncertainty was also raised responses to V1 and V3. Half of the responders were aware of dental services which could treat dental trauma and minority of responders eluded to the need for follow up.

Confidential: For Review Only

Access

When asked if the ED was an appropriate environment for paediatric dental trauma to present, almost half(n=45) thought that is appropriate; whilst n=16 disagreed, and n=33 were unsure.

A common theme raised was the need to improve dental teaching amounst the paediatric emergency medical team, leading some responders to conclude that the PED is not an appropriate place for a dental trauma to present. Proposed improvements included an easy-to-use decision-making tool, regular training of staff including online resources, and suggestions that dental trauma should be included in the medical undergraduate curriculum.

> *'Doctors have almost no dental training as part of their course and there is little teaching available currently. My decision above would change if we had teaching and a decision-making tool…'*

Many responders commented how access depends on the configuration of local services. They noted that ED attendance would be appropriate if there was a structured urgent referral service for dental trauma, including improved communication with dental colleagues. Other suggestions to improve management included establishing emergency dental networks and *'more streamlined community dental services, in particular staff urgent and emergency dentists with professionals who are comfortable in dealing with children.'*

Numerous responders concluded that the PED was the most appropriate place because of a '*deficit of community dental provision…'* to treat paediatric dental trauma. One responder added: *'It is not the best place for management but often the only option for patients- most dental trauma presents at weekends or out of hours'.* There was acknowledgment that dental hospitals can provide complex emergency dental treatment in working hours but paediatric dental services were limited out of hours.

Confidential: For Review Only

Responders commented that if the injury was localised to the teeth, it would be more appropriate to attend a GDP, not the PED. However, many responders noted that children are often referred to ED by GDPs. Some responders recognised that management of injuries, such as avulsion requiring re-implantation, is time critical and therefore a PED should be able to manage this and it is appropriate.

> *'Depends on the mechanism of injury and any concurrent (non-dental) injuries. For isolated dental trauma, GDP is far more appropriate.'*

It was suggested that more information and resouces should be available to signpost parents where to take their child if they have dental trauma because '*not many parents are aware they can take children to the dental hospital and therefore wait a long time in ED before they are referred to the dental or max fax team.'* It was suggested that resources should include *'clear information for families (in multiple languages) about home first aid for dental trauma, and the most appropriate resources within the region'.*

DISCUSSION

This study provides insight into emergency department health professionals' experience, perceptions, awareness and confidence in managing TDIs specifically within a Paediatric Emergency Department setting, addressing both primary and secondary objectives.

TDIs are more common than childhood asthma, yet it is often overlooked in medical and nursing curriculums, as well as within ED education programmes. It is widely reported that medical clinicians' knowledge of TDI management is lacking, this study demonstrates how an overwhelming number of responders identified the need for more TDI teaching, with only 12 responders reporting that they received some form of teaching on dental trauma as part of their undergraduate studies, comparable to the findings from studies undertaken in various countries. [12-19 22]

Responders' awareness of how to recognise and manage TDIs was markedly varied. It is noted that TDIs are not life threatening, however, the results of a dental trauma can cause undue psychological

Confidential: For Review Only

affects throughout childhood, in addition they can potentially cause undesirable dental consequences. Therefore, it is important for all ED clinicians to have an awareness of TDI recognition and management. There was an overall lack of awareness of the need of TDI follow up, which to the authors knowledge, has not been reported in previous published studies. Parents should be made aware of the potential complications and the importance of regular recalls with their dentist. Furthermore, there was little awareness of need for splinting luxation injuries, taking radiographs, and a minority of responders displayed awareness of potential non-accidental injuries (NAI) associated with TDIs. Hence, there is scope to improve the dental teaching received as part of the UK undergraduate curriculum in medicine and within nursing training programme and other associated healthcare training. This supports recent proposals to develop an online dental trauma course for medical professionals. [23 24]

A common theme raised by responders, was confusion whether the injured teeth in the vignettes were primary or permanent teeth. The age stated in the vignette question could have informed the clinician whether it was permanent tooth, supporting findings that ED health professionals' lack knowledge of tooth eruption ages.[22 25] Presentation and management of TDIs in children is notably different to adults. Paediatric TDIs may involve immature anterior permanent teeth, and cooperation can vary. Thus, understanding eruption ages of permanent teeth is of great significance. There is an argument that depending on the specialty, medical clinicians may not require dental trauma knowledge. However, dental trauma could readily present to EDs, GPs, it can be caused by intubation by anaesthetic teams, and could happen to any patient under any medical specialty.

ED health professionals' confidence in assessing TDIs was notably low, comparable to a previous study.[26] However, this study assessed all health professionals who may encounter dental trauma in the PED, not just paediatric emergency physicians, finding that seniority is not directly associated with confidence and knowledge, opposing the results from another study.[25]

https://mc.manuscriptcentral.com/bmjpo

Confidential: For Review Only

Many health professionals raised concerns that there is a lack of emergency dental services in their regions within the UK, resulting in many families choosing to attend a PED, echoing findings of a previous study.[25] The two major children's units within this study both have OMFS oncall and demonstrated an element of dependency towards oncall OMFS staff. However, many district hospitals do not have this configuration and require an onwards referal to larger regional units with an oncall dentally qualified clinician. This may not only be inconvenient for the child and their family, but also increases the time between injury and management, impacting the prognosis of the tooth.

Responders suggested improving signposting for families and the need for a universal TDI decision-making-tool. This could be utilised by all dental and non-dental health professionals to identify the urgency of the TDI and direct children to the best place for management. In order to improve the current services in the UK, more research is required to understand the use of available services. Managed clinical networks (MCNs) could help advance the services available.[27]

It had been acknowledged that there are limitations to this study. Overall response rate was 63% which is not uncommon for a research survey. However, a higher response rate would have been beneficial. Despite this, the cohort of responders represented a range of ED health professionals who appropriately addressed the aim. Response bias cannot be excluded; those more interested, competent, or confident in management of TDIs are more likely to respond to the survey. In order to be more generalisable, it would be preferential to extend the survey to other PEDs in the UK but it should be noted that AHH and BCH cover a large proportion of the North West and Midlands, England, and the staff have been trained in a variety of units across the country.

CONCLUSIONS

Confidential: For Review Only

Health professionals within two large regional PEDs in the UK recognise that there is a need to enhance dental trauma teaching for emergency department clinicians who regularly encounter TDIs, to increase their confidence and enable them to triage and advise families appropriately.

An improved pathway between dental and emergency services within these regions of the UK is required for TDI patients and it would be beneficial to improve sign posting for families, and create a decision-making tool for non-dental clinicians to ensure children with a TDI receive the required management and follow up. This could, in turn, improve outcomes and experience for children who experience a TDI.

Further research should be undertaken to help improve the emergency management of TDIs in children within the UK.

ETHICS STATEMENT

Ethical approval was obtained by the Health and Life Sciences Research Ethics Committee, University of Liverpool, reference number 7585.

Patient and public involvement statement

There was no patient or public involvement in this study

COMPETING INTEREST

None declared

ACKNOWLEDGMENTS

Thank you to the staff at Alder Hey Children's Hospital and Birmingham Children's Hospital Emergency Departments who frequently manage dental patients and kindly participated in this study.

FUNDING STATEMENT

Confidential: For Review Only

This research received no specific grant from any funding agency in the public, commercial or not-for-profit sectors.

CONTRIBUTIONS

N Gallichan: Conceptualisation, methodology, investigation, data curation, writing- original draft preparation. **S Albadri**: Conceptualisation, methodology, writing- reviewing and editing, visualisation and supervision. **F Watkins**: Methodology, writing- reviewing and editing, investigation. **F Jarad:** Methodology, supervision. **S Messahel**: Conceptualisation, project administration, writing-reviewing and editing. **S Hartshorn**: Project administration, writing- reviewing and editing. **L Gartshore:** Conceptualisation, methodology, supervision**,** writing- reviewing and editing, visualisation

DATA SHARING

Data is available on request

REFERENCES

1. Heggie C, Gallichan N, Gartshore L, et al. Traumatic and non-traumatic dental presentations to the Paediatric Emergency Departments of two UK Children's Hospitals: a multi-centre evaluation. *International Journal of Paediatric Dentistry* 2021;32(1):90-100.2. Marshman Z, Broomhead T, Rodd HD, et al. Who attends a Children's Hospital Emergency Department for dental reasons? A two-step cluster analysis approach. *Community Dent Oral Epidemiol* 2017;45(1):49-58. doi: 10.1111/cdoe.12258 [published Online First: 2016/09/30]
3. Andreasen J, Ravn J. Epidemiology of traumatic dental injuries to primary and permanent teeth in a Danish population sample. *International journal of oral surgery* 1972;1(5):235-39.
4. Hamilton FA, Hill FJ, Mackie IC. Investigation of lay knowledge of the management of avulsed permanent incisors. *Dental Traumatology* .1997;13(1):19-23..
Hamilton FA, Hill FJ, Mackie IC. Investigation of lay knowledge of the management of avulsed permanent incisors.. 1997 Feb
5. Chadwick BL, White D, Morris A, et al. Non-carious tooth conditions in children in the UK, 2003. *British Dental Journal* 2006;200(7):379.
6. O'Brien M. Children's dental health in the United Kingdom 1993: HM Stationery Office; 1994.7. Petti S, Glendor U, Andersson L. World traumatic dental injury prevalence and incidence, a meta-analysis-One billion living people have had traumatic dental injuries. *Dental Traumatology* 2018;34(2):71-86. doi: 10.1111/edt.12389
8. Welbury R, Duggal MS, Hosey MT. Paediatric dentistry: Oxford university press 2018.
9. Abbott B, Zybutz C, Scott KM, et al. A review of the hours dedicated to oral health education in medical programmes across Australia. *Internal Medicine Journal* 2018;48(9):1035-40. doi: 10.1111/imj.14021

Confidential: For Review Only

10. Aren A, Erdem AP, Aren G, et al. Importance of knowledge of the management of traumatic dental injuries in emergency departments. *Ulus Travma Acil Cerrahi Derg* 2018;24(2):136-44.
11. RCEM C. Paediatric Emergency Medicine, Sub-specialty Syllabus. [online] 2021. https://www.rcpch.ac.uk/sites/default/files/2021-09/Appendix%204M%20-%20Paediatric%20Emergency%20Medicine.pdf (accessed 3rd November 2021)
12. Abu-Dawoud M, Al-Enezi B, Andersson L. Knowledge of emergency management of avulsed teeth among young physicians and dentists. *Dental Traumatology* 2007;23(6):348-55.
13. Bahammam LA. Knowledge and attitude of emergency physician about the emergency management of tooth avulsion. *BMC oral health* 2018;18(1):1-9.
14. Holan G, Shmueli Y. Knowledge of physicians in hospital emergency rooms in Israel on their role in cases of avulsion of permanent incisors. *International Journal of Paediatric Dentistry* 2003;13(1):13-19.
15. Ulusoy AT, ÖNDER H, Cetin B, et al. Knowledge of medical hospital emergency physicians about the first-aid management of traumatic tooth avulsion. *International journal of paediatric dentistry* 2012;22(3):211-16.
16. Yigit Y, Helvacioglu-Yigit D, Kan B, et al. Dentofacial traumatic injuries: A survey of knowledge and attitudes among emergency medicine physicians in Turkey. *Dental Traumatology* 2019;35(1):20-26.
17. Subhashraj K. Awareness of management of dental trauma among medical professionals in Pondicherry, India. *Dental Traumatology* 2009;25(1):92-94.
18. Zencircioglu OK, Eden E, Ocek ZA. Access to health care after dental trauma in children: A quantitative and qualitative evaluation, 2019:163-70.
19. Trivedy C, Kodate N, Ross A, et al. The attitudes and awareness of emergency department (ED) physicians towards the management of common dentofacial emergencies. *Dental Traumatology* 2012;28(2):121-26.
20. Sharma A, Minh Duc NT, Luu Lam Thang T, et al. A consensus-based checklist for reporting of survey studies (CROSS). *Journal of general internal medicine* 2021;36(10):3179-87.
21. Dental Trauma Guide Website: Resource Centre for Rare Oral Diseases and Department of Oral and Maxillo-Facial Surgery at the University Hospital of Copenhagen; [online] 2021. https://dentaltraumaguide.org/. (accessed 13th October 2022)
22. Patel K, Driscoll P. Dental knowledge of accident and emergency senior house officers. *Emergency Medicine Journal* 2002;19(6):539-41.
23. Yeng T, O'Sullivan AJ, Shulruf B. A Proposal to Introduce Dental Trauma into Medical Education: An Insight. *Dental Traumatology* 2020; 36(4):390-2.24. Yeng T, O'Sullivan AJ, Shulruf B. Online dental trauma course for medical education. *Dental Traumatology.* 2021;37(6):803-6.25. Samaei H, Weiland TJ, Dilley S, et al. Knowledge and confidence of a convenience sample of Australasian emergency doctors in managing dental emergencies: results of a survey. *Emergency medicine international* 2015;4:2015.
26. Cully M, Cully J, Nietert PJ, et al. Physician Confidence in Dental Trauma Treatment and the Introduction of a Dental Trauma Decision-Making Pathway for the Pediatric Emergency Department. *Pediatric Emergency Care* 2019;35(11):745-48. doi: 10.1097/PEC.0000000000001479
27. NHS England. Commissioning Standard for Dental Specialties – Paediatric Dentistry. Leeds. [online] 2018. https://www.england.nhs.uk/wp-content/uploads/2018/04/commissioning-standard-for-dental-specialties-paediatric-dentristry.pdf (accessed 6th December 2022).

Confidential: For Review Only

Confidential: For Review Only

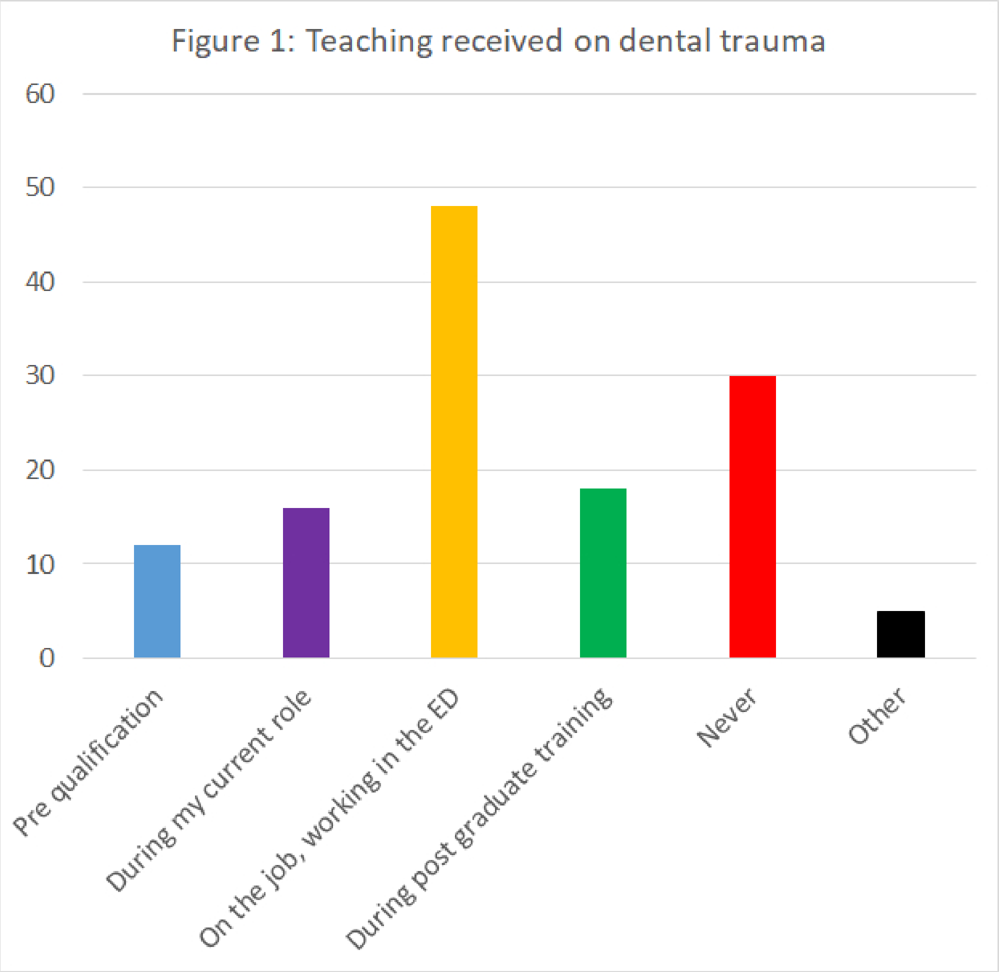

Figure 1: Teaching on dental trauma

109x106mm (144 x 144 DPI)

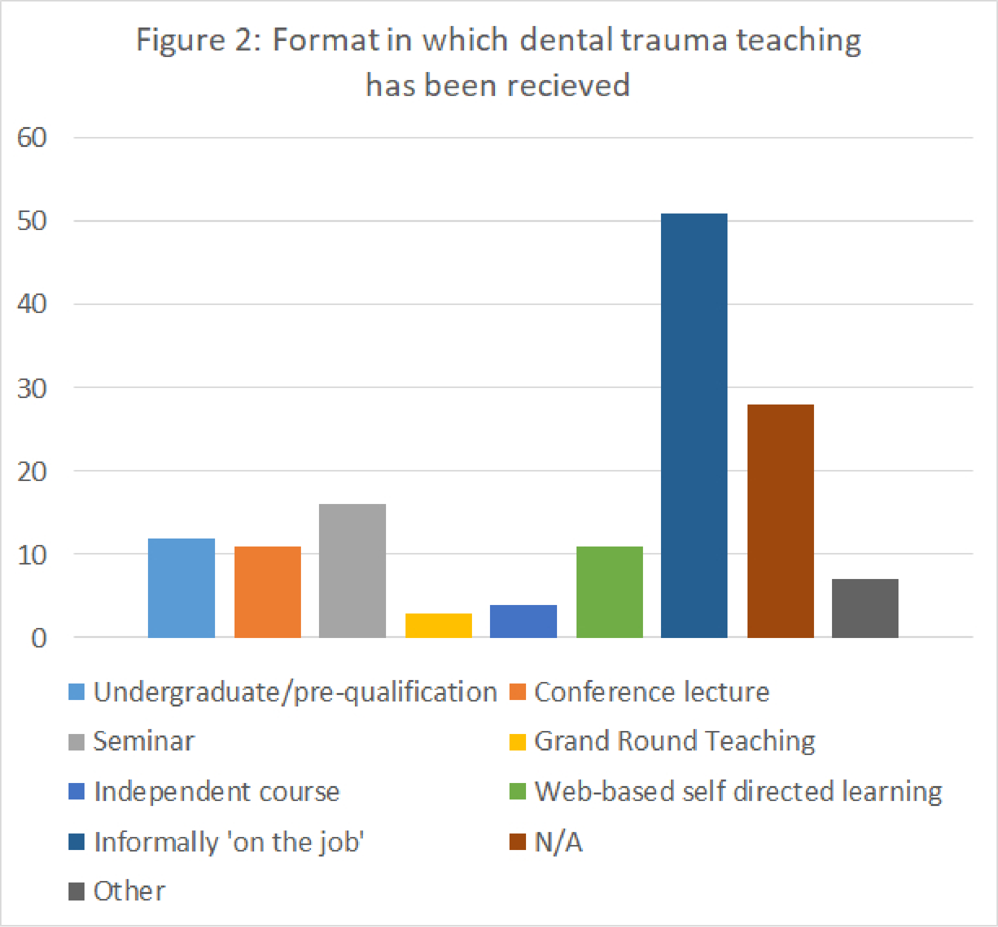

Figure 2: Format in which dental trauma teaching has been received

108x100mm (144 x 144 DPI)

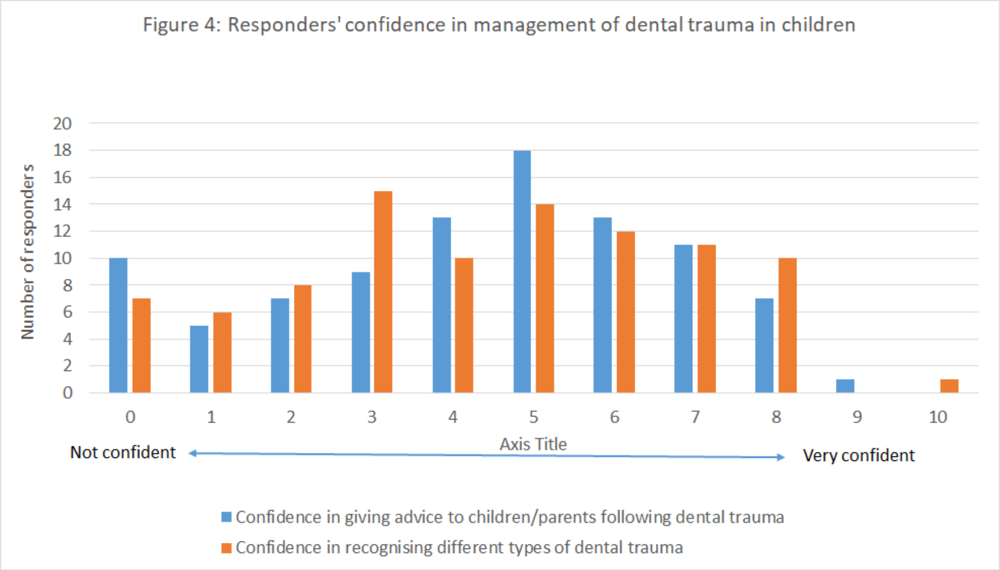

Figure 3: responders confidence

174x99mm (144 x 144 DPI)

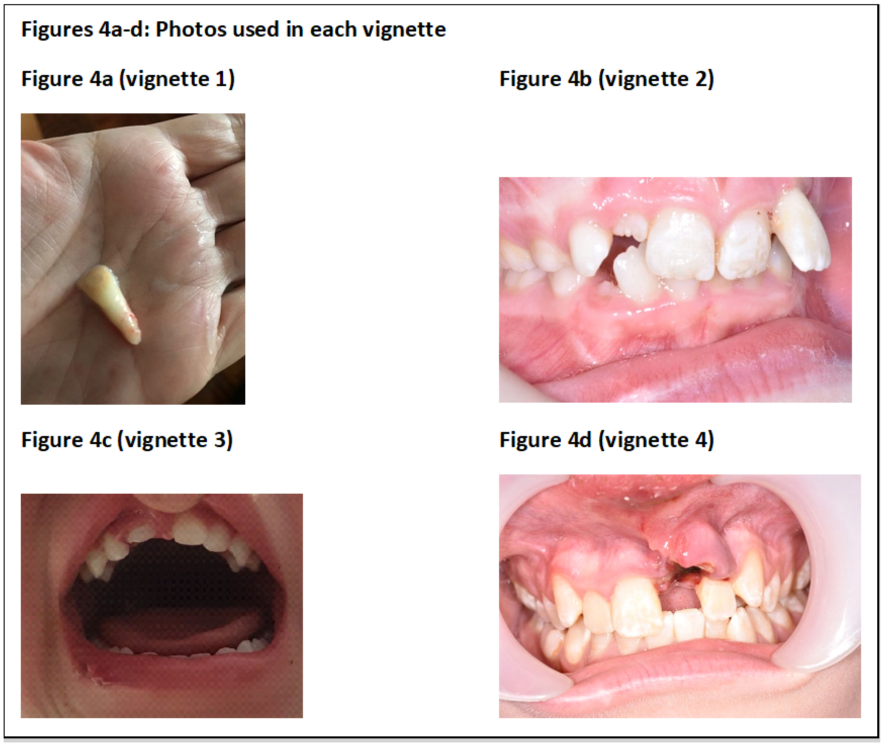

Figures 4a-d: Photos used in vignettes 1-4

174x132mm (144 x 144 DPI)
