# Supplementary material for: Management of traumatic dental injuries: a survey of paediatric emergency department health professionals
Source: BMJ Paediatr Open. 2023 Mar 22;7(1):e001740. doi: 10.1136/bmjpo-2022-001740 (PMC10040074; doi:10.1136/bmjpo-2022-001740)
Supplement: Reviewer comments [file bmjpo-2022-001740.reviewer_comments.pdf]

PEER REVIEW HISTORY

BMJ Paediatrics Open publishes all reviews undertaken for accepted manuscripts. Reviewers are asked to complete a checklist review form and are provided with free text boxes to elaborate on their assessment. These free text comments are reproduced below.

ARTICLE DETAILS

**TITLE (PROVISIONAL)**	Management of traumatic dental injuries: a survey of Paediatric Emergency Department health professionals
**AUTHORS**	Gallichan, Nathalie; Albadri, Sondos; Watkins, Francine; Jarad, Fadi; Messahel, Shrouk; Hartshorn, Stuart; Gartshore, Laura

VERSION 1 - REVIEW

**REVIEWER**	Reviewer Name: Waterfield, Thomas<br>Affliation: Wellcome Wolfson Institute for Experimental Medicine, Queen's University Belfast, Belfast BT97BL, UK<br>Conflict of Interest: None
**REVIEW RETURNED**	10-Nov-2022

**GENERAL COMMENTS**	Thank you for giving me the opportunity to review this manuscript assessing Paediatric Emergency Department (PED) clinicians' confidence, experience and<br>awareness in managing traumatic dental injuries (TDIs).<br><br>The study is an online questionnaire conducted at two English Children's Hospitals with responses from 94 clinicians across a range of settings. Of these only 1/4 had experience of managing dental trauma regularly in the ED (n=28%). The authors report a lack of training for managing dental trauma and significant variation in clinician experience and expertise.<br><br>The authors conlcude that all EDs that encounter dental trauma require greater training.<br><br>The survey is well constructed, the manuscript well written.<br><br>My major concern is that more sites should be included to make the findings more reliable and generalisable. I would advise that the authors consider approaching a resrearch network and expanding the s

**REVIEWER**	Reviewer Name: Dr. Michael Barrett<br>Affliation: Children's Health Ireland, Emergency Medicine<br>Conflict of Interest:None
**REVIEW RETURNED**	21-Nov-2022

**GENERAL COMMENTS**	Thanks you for the opportunity to review,<br><br>I believe this article merits publication and is of interest.<br><br>I'm utilising the Equator CROSS checklist for the reporting of Survey Studies to assist the review. The word 'survey' seems to be

missing from the abstract or title. Can the authors explain or address?

A STROBE checklist was used in the methodology section. Can the authors please explain why this approach was taken for a survey study instead of a Equator recommended CHERRIES or CROSS checklist?
I believe this study to be a cross-sectional survey study delivered electronically. Please explain if this is not the case.

In the abstract, I suspect owing to the study design, the first sentence in the conclusion cannot be stated as such. Surveying 2 large regional PEDs with an on call dental service does not reflect the experience of all emergency clinicians but only those working the PEDs alone. surveyed. This survey data could reflect the national reality, it may under represent the reality or over represent the reality. We don't yet know. This survey could be easily expanded to include a representative sample of facilities to make it generalisable.

Introduction: The first sentence : please provide a reference. Where does it comes from ?

Methods:

Where did the target sample size originate from?

Please consider including the actual survey in the appendices for reference.

This is a cross-sectional survey design and this wording should be considered for inclusion.

Please better describe the NPS scale used in the methodology

Report the method of pretesting the survey/questionnaire, number of times it was pre-tested, reported validity and reliability information and reference links.

What were the inclusion criteria and exclusion criteria for the study population? This is important and also leads me to the question: Table 1: is a health care assistant a clinician? If not they should be excluded and this should be mentioned. How did they get asked if they are not clinicians?

I believe this was a convenience sample of respondents please confirm and if so include in the methodology.

Can you provide a statement on survey anonymity and confidentiality of respondents ?

Describe the statistical methods and analytical approach with the software used.

How was missing data handled?

With respect to the qualitative results under what method (provide a reference) was the thematic analysis conducted? Was there software involved?

	Results section<br><br>May I enquire would it be expected that nursing staff and healthcare attendants would utilise a decision-making pathway in their daily practice?<br>My impression that it would not be standard practice and ,if this is true, this section of the survey is not a fair reflection of their daily practice. This will need to be considered in the presentation of the results (this subgroup of respondents may need exclusion for this section)<br><br>I may have missed this explain the acronym OMFS.<br><br>Pg 8 line 30: consider using the term 'PEM (paediatric emergency medicine) team' instead of 'medical team' if that is what you mean. Or perhaps you mean community understanding or what is appropriate or inappropriate to refer. This is not clear. Perhaps a referenced qualitative statement might better explain what is meant here.<br><br>Discussion : pg 11 line 42-43 please reference the statement about MCNs.<br><br>The response rate stated in the discussion needs to be stated in the first paragraph of the results section. It must not be introduced in the discussion without being referenced from the results section.<br><br>Pg 11/12 56-57/3-5 : I do not agree that you can state that these are generalisable. Yes they are regional but the majority of children with dental injuries may not be attending a PED of a tertiary paediatric hospital with an on call dental service. Also if there is regional variation of the out of hours dental services outside of the PED /emergency dept you have not account for this. Please consider revising this sentence taking special note of the sample bias and how it does not represent the national picture which I'm varies from region to region.<br><br>Pg 12 Conclusion line one : I believe you mean 'PEM clinicians' not 'medical clinicians'. please correct.<br><br>I'm not sure why the strobe list is included in the main manuscript. Checklists of items may be included in the appendix and consider the type of checklist in light of my previous comments.<br><br>References : 1 and 23 are incomplete.<br><br>Figure 4 : Axis title is missing<br><br>Figures 5 a-d: This may be further enhanced/improved by providing the vignette of each case.<br><br>Well done. We look forward to seeing the revised manuscript

VERSION 1 – AUTHOR RESPONSE

Reviewer: 1

Dr. Thomas Waterfield

Thank you for giving me the opportunity to review this manuscript assessing Paediatric Emergency Department (PED) clinicians’ confidence, experience and

awareness in managing traumatic dental injuries (TDIs).

The study is an online questionnaire conducted at two English Children's Hospitals with responses from 94 clinicians across a range of settings. Of these only 1/4 had experience of managing dental trauma regularly in the ED (n=28%). The authors report a lack of training for managing dental trauma and significant variation in clinician experience and expertise.

The authors conclude that all EDs that encounter dental trauma require greater training.

The survey is well constructed, the manuscript well written.

My major concern is that more sites should be included to make the findings more reliable and generalisable. I would advise that the authors consider approaching a research network and expanding the sites.

Thank you for your summary and comments. We appreciate the advice, including more sites is certainly desirable. Unfortunately, due to the timelapse and pressures /changes to access we believe this may create bias.

However future work will reach out to more centres and utilise a research network such as PERUKI. We do however feel that this is still a good representative sample of the population, the two children’s hospital included are the hub children’s hospitals across the North West of England, North Wales (North- South from Carlisle to Crewe; West-East North Wales to East Cheshire inclusive), the Midlands and Mid Wales (West - East from mid-Wales to Coventry, North - South from Stafford to Cheltenham). They cover a very large area, and outside of London they are the two largest PEDs in the UK. Furthermore, the health professional’s medical education/training could have been undertaken anywhere in the country, this is not restricted to these two areas.

Reviewer: 2

Dr. Michael Barrett, Children's Health Ireland, University College Dublin

Thanks you for the opportunity to review,

I believe this article merits publication and is of interest.

I'm utilising the Equator CROSS checklist for the reporting of Survey Studies to assist the review. The word 'survey' seems to be missing from the abstract or title. Can the authors explain or address? Addressed, this was an oversight and we agree on your comments.

A STROBE checklist was used in the methodology section. Can the authors please explain why this approach was taken for a survey study instead of an Equator recommended CHERRIES or CROSS checklist? Addressed. This is an observational study and originally when I was uploading the manuscript, STROBE seemed the most appropriate from the list on the BMJ submissions guidelines as it is cross-sectional. We thank you for bringing this up because CROSS or CHERRIES should be used and have therefore replaced the STROBE checklist with CROSS.

I believe this study to be a cross-sectional survey study delivered electronically. Please explain if this is not the case. Addressed, we agree. thank you.

In the abstract, I suspect owing to the study design, the first sentence in the conclusion cannot be stated as such. Surveying 2 large regional PEDs with an on call dental service does not reflect the experience of all emergency clinicians but only those working the PEDs alone. surveyed. This survey data could reflect the national reality, it may under represent the reality or over represent the reality. We don't yet know. This survey could be easily expanded to include a representative sample of facilities to make it generalisable. We agree. I have amended the wording of the first sentence of the conclusion.

Introduction: The first sentence: please provide a reference. Where does it comes from ? Addressed, thank you, I moved the references which are the relevant ones, from the following sentence to be cited after the first sentence.

Methods:

Where did the target sample size originate from? Total number of health professionals who could assess TDIs within each of the PEDs.

Please consider including the actual survey in the appendices for reference. We have considered this and thank you for your comment, we have attached to be included in appendices if required.

This is a cross-sectional survey design and this wording should be considered for inclusion. This was included in the first sentence of the study design.

Please better describe the NPS scale used in the methodology Addressed, thank you.

Report the method of pretesting the survey/questionnaire, number of times it was pre-tested, reported validity and reliability information and reference links.

I have added a summary description of the method in the survey design section.

Pre-testing was completed and served to improve clarity, and that the intention of the investigator was correctly understood. Pilot testing was also completed and served to edit the content of the questionnaire items to the agreed preferences of the pilot participants. Content and face validity were assessed during the pre-test and pilot phases. Efforts were made to evaluate whether the questionnaire measured what it was intended to measure during these processes. However, the comprehensiveness, reliability and validity of this questionnaire would be further enhanced by clinical sensibility testing. Clinical sensibility testing would serve to determine that this novel questionnaire answers the research question and the objectives of the survey.

Reliability testing of this questionnaire for future use might incorporate test-retest reliability to provide statistical analysis of whether the same question posed to the same individual yields consistent results at a four-week interval, providing intra-rater reliability. Inter-rater reliability is perhaps of less importance that test-retest reliability in the case of this survey.

Assessment of internal consistency would enable understanding of whether or not different question items that tap into the same domains or constructs are correlated. In this survey, this might have been particularly useful in relation to the construct of clinical decision-making practice which was investigated by the vignettes.

What were the inclusion criteria and exclusion criteria for the study population? This is important and also leads me to the question: Table 1: is a health care assistant a clinician? If not they should be excluded and this should be mentioned. How did they get asked if they are not clinicians? Addressed, included the inclusion and exclusion criteria. With regards to your second question, a health care assistant can still provide advice and supports the rest of the team. They hold a clinical role and therefore we would consider them as a clinician. Following the comments above, we appreciate how the term clinician may be interpreted as medics, but our intention was to use a term which we believed to encompass anyone in the ED who may clinically encounter a child with a TDI. We hope the suggested term of ED health professional is more acceptable.

I believe this was a convenience sample of respondents please confirm and if so include in the methodology. Addressed in the design section thank you.

Can you provide a statement on survey anonymity and confidentiality of respondents? Addressed under in ‘survey design, sample and distribution’, first paragraph, first sentence.

Describe the statistical methods and analytical approach with the software used. We engaged with a statistician when writing the protocol, they confirmed that the results would require descriptive statistics. A comment has been added to under ‘survey design and content’ section. Thank you.

How was missing data handled? Addressed, there was no missing data, a comment was added to the der ‘survey design and content’ section. Thank you.

With respect to the qualitative results under what method (provide a reference) was the thematic analysis conducted? Was there software involved? Thematic analysis was conducted by coding the answers and using reflexive thematic analysis to find themes, capturing a shared meaning, united by a central organising concept (Braun & Clarke 2022). We did not use any software.

Results section

May I enquire would it be expected that nursing staff and healthcare attendants would utilise a decision-making pathway in their daily practice?

My impression that it would not be standard practice and ,if this is true, this section of the survey is not a fair reflection of their daily practice. This will need to be considered in the presentation of the results (this subgroup of respondents may need exclusion for this section)

Nursing staff do utilise a decision making pathway in their daily practice – this is done when triaging patients on arrival to the ED and allocating acuity. The health care assistant would not however, but there is only one of them in the respondents. They are however in a position to give advice and provide care to patients in the ED. The ED guidelines (local handbook) is not just for doctors, it is for nursing staff too. The minor injuries pathways are a combination of identification/ diagnosis/ intervention/ follow up pathways, and often have instruction for nursing staff on type of splint or dressing to be used.

I may have missed this explain the acronym OMFS. Addressed and used full term when first used in the summary of themes in results section, thank you.

Pg 8 line 30: consider using the term 'PEM (paediatric emergency medicine) team' instead of 'medical team' if that is what you mean. Or perhaps you mean community understanding or what is appropriate or inappropriate to refer. This is not clear. Perhaps a referenced qualitative statement might better explain what is meant here. Addressed, using the term PEM team. Thank you.

Discussion: pg 11 line 42-43 please reference the statement about MCNs. Addressed by adding the reference, thank you.

The response rate stated in the discussion needs to be stated in the first paragraph of the results section. It must not be introduced in the discussion without being referenced from the results section. Addressed, thank you.

Pg 11/12 56-57/3-5 : I do not agree that you can state that these are generalisable. Yes they are regional but the majority of children with dental injuries may not be attending a PED of a tertiary paediatric hospital with an on call dental service. Also if there is regional variation of the out of hours dental services outside of the PED /emergency dept you have not account for this. Please consider revising this sentence taking special note of the sample bias and how it does not represent the national picture which I'm varies from region to region. Addressed. Thank you.

Pg 12 Conclusion line one : I believe you mean 'PEM clinicians' not 'medical clinicians'. please correct. Addressed. Thank you.

I'm not sure why the strobe list is included in the main manuscript. Checklists of items may be included in the appendix and consider the type of checklist in light of my previous comments. Addressed, thank you.

References: 1 and 23 are incomplete. Addressed, thank you- other references have also been checked.

Figure 4 : Axis title is missing Addressed- figure 4 has been removed.

Figures 5 a-d: This may be further enhanced/improved by providing the vignette of each case. Addressed. The vignette of each case has been provided. Thank you.

Well done. We look forward to seeing the revised manuscript

VERSION 2 – REVIEW

**REVIEWER**	Reviewer Name: Dr. Michael Barrett<br>Affliation: Children's Health Ireland, Emergency Medicine<br>Conflict of Interest:None
**REVIEW RETURNED**	18-Jan-2023

**GENERAL COMMENTS**	Congratulations on the revised manuscript and your responses. It reads as a well-balanced informative piece of work.<br><br>I spotted a slight written error in Figure 3 with the title in figure 3 stating it is figure 4. There is a mismatch between the figure titles written. The words 'axis title' should be removed.<br><br>Otherwise all good. Well done.
